# Transcriptome Analysis of Tolerant and Susceptible Maize Genotypes Reveals Novel Insights about the Molecular Mechanisms Underlying Drought Responses in Leaves

**DOI:** 10.3390/ijms22136980

**Published:** 2021-06-29

**Authors:** Joram Kiriga Waititu, Xingen Zhang, Tianci Chen, Chunyi Zhang, Yang Zhao, Huan Wang

**Affiliations:** 1 Biotechnology Research Institute, Chinese Academy of Agricultural Sciences, Beijing 100081, China; joram.kiriga@gmail.com (J.K.W.); zhangchunyi@caas.cn (C.Z.); 2 National Engineering Laboratory of Crop Stress Resistance Breeding, School of Life Sciences, Anhui Agricultural University, Hefei 230036, China; 13145518767@163.com (X.Z.); chentianci1996@163.com (T.C.); 3 National Agricultural Science and Technology Center, Chinese Academy of Agricultural Sciences, Chengdu 610213, China

**Keywords:** maize, drought stress, transcriptome, RNA sequencing, lncRNA

## Abstract

Maize (*Zea mays* L.) is the most essential food crop in the world. However, maize is highly susceptible to drought stress, especially at the seedling stage, and the molecular mechanisms underlying drought tolerance remain elusive. In this study, we conducted comparative transcriptome and physiological analyses of drought-tolerant (CML69) and susceptible (LX9801) inbred lines subjected to drought treatment at the seedling stage for three and five days. The tolerant line had significantly higher relative water content in the leaves, as well as lower electrolyte leakage and malondialdehyde levels, than the susceptible line. Using an RNA-seq-based approach, we identified 10,084 differentially expressed genes (DEGs) with 6906 and 3178 DEGs been annotated and unannotated, respectively. Two critical sets of drought-responsive DEGs, including 4687 genotype-specific and 2219 common drought-responsive genes, were mined out of the annotated DEGs. The tolerant-line DEGs were predominantly associated with the cytoskeleton, cell wall modification, glycolysis/gluconeogenesis, transport, osmotic regulation, drought avoidance, ROS scavengers, defense, and transcriptional factors. For the susceptible line, the DEGs were highly enriched in the photosynthesis, histone, and carbon fixation pathways. The unannotated DEGs were implicated in lncRNAs, including 428 previously reported and 22% putative TE-lncRNAs. There was consensus on both the physiological response and RNA-seq outcomes. Collectively, our findings will provide a comprehensive basis of the molecular networks mediating drought stress tolerance of maize at the seedling stage.

## 1. Introduction

Drought is one of the most important abiotic stresses threatening worldwide agricultural production and food safety [1,2,3]. On average, it reduces global cereal production by 10.1% and affects 64% of the worldwide land area [4,5]. Surprisingly, current global climate change models are forecasting more frequent and severe weather events along with an overall temperature rise [6]. As a result, water scarcity is anticipated to worsen, with a much more significant impact on the physiological status and productivity of major crops expected in the coming decades [7]. Thus, understanding drought tolerance mechanisms in crops and developing drought-tolerant varieties is critical for maintaining crop yields under drought conditions.

Maize (*Zea mays* L.) is an important food crop surpassing rice and wheat since 2012 [8]. It is a versatile cereal crop that is highly susceptible to drought stress at all stages of development [9]. Previous studies have shown that maize yield decreases by 10–76% depending on the severity of the water stress, the susceptibility of the crop, and the stage of production [10]. Generally, the maize seedling stage requires less water compared to advanced vegetative and anthesis development stages [11]. Nevertheless, the moisture deficit in the seedling stage will hamper both the early crop establishment and the entire growth cycle, thereby affecting the plant adaptive capacity at an early stage and reducing the yield potential [12]. Thus, elucidating the mechanism of how maize responds to drought stress at the seedling stage has great significance for maize production as well as for the maize breeding programs.

In plants, drought stress induces an extensive range of responses such as increasing oxidative damage in the chloroplast, inhibiting photosynthesis, restricting metabolic reactions, activating sugar catabolism, and modifying the composition of cellular lipid [13,14,15,16,17]. However, plants have developed various strategies to cope with stress through a complicated and coordinated response involving physiological and metabolic reprogramming, transcription regulation, epigenetic, and the expression and interaction of thousands of genes with multiple environmental variables throughout the plant developmental cycle [18,19]. Normally, once the plant is subjected to drought stress, the stress stimuli are perceived through alterations in turgor pressure or activities of membrane receptors. These extracellular signals are then transformed into intracellular signals by generating second messengers such as calcium ions, inositol phosphate, and nitric oxide [20,21]. These second messengers subsequently initiate the corresponding signal transduction pathways mediated by protein kinases and phosphatases, which activate or suppress transcription factors (TFs). The TFs are then regulated by other upstream components at the transcription level and modified at the post-transcription level through ubiquitination and sumoylation, thereby forming a dynamic regulatory network that regulates the expression of stress-responsive genes [22]. The complex gene expression cascades activated in turn determine the activation of physiological and metabolic responses through the generation of larger and deeper root systems [23], regulation of stomatal closure via abscisic acid (ABA) to reduce leaves water loss [24], accumulation of osmoprotectants such as amino acids, glycine betaine, and sugars, which are vital for osmotic adjustments [25]. Moreover, there is an enhancement of protective protein such as late embryogenesis abundance (LEA) [26] and an increase in the level of antioxidants systems [27]. All these responses involve multiple biochemical pathways and significant changes in gene expression. Thus, the identification of specific genes and pathways associated with drought tolerance is a fundamental advance in the improvement of drought-tolerant varieties.

Although significant scientific breakthrough has been made in deciphering drought response mechanism between distinct inbred lines at the seedling stage [28,29], there remains an inadequate understanding of the molecular mechanisms and genes involved in mediating drought response at the seedling stage of maize [30]. However, the advancement of next-generation sequencing, RNA sequencing (RNA-seq), has enabled researchers to decipher transcriptome analyses of plants’ drought stress response [31]. The reasonable cost, high throughput, and sensitivity of RNA-seq [32] have enhanced vast knowledge regarding gene expression networks that modulate drought response in various plants such as maize [19], barley [33], cotton [34], and rice [35], thereby aiding in breeding better-adapted crop species. In the current study, the understanding of drought stress response at the seedling stage was further extended through transcriptomic analysis of drought-tolerant (CML69) and drought-susceptible (LX9801) inbred lines under different drought conditions. Massive parallel sequencing of RNA-seq under control, three days, and five days drought treatment were used on the Illumina HiSeq sequencing platform to provide an in-depth transcriptome scenario of the two inbred lines in response to drought stress. The resulting transcriptome data were then used to pinpoint specific genes and pathways that could be involved in drought stress response and to clarify the possible molecular processes involved in maize adaptation to the distinct magnitude of drought stress. Our findings will enhance the grasp of drought-tolerant mechanisms in maize at the seedling stage and will serve as an invaluable molecular-level reference to inform future studies on improving drought tolerance in maize.

## 2. Results

### 2.1. Morphological and Physiological Analysis of CML69 and LX9801 Seedlings in Responses to Drought Stress

**Figure 1:**
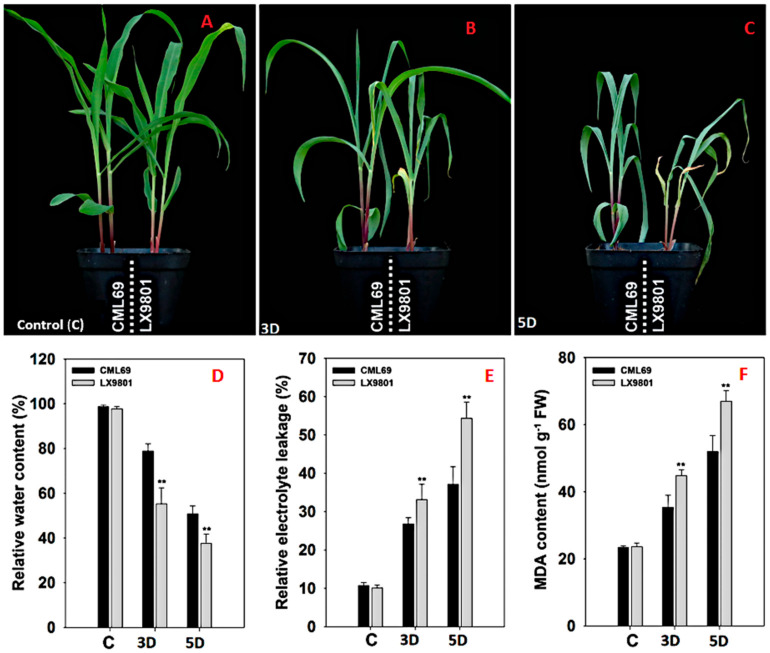
Phenotypic and physiological responses of drought-tolerant line CML69 and drought-susceptible line LX9801. The seedling phenotypic response of CML69 and LX9801 at; (**A**) control (C)-well-watered plants; (**B**) three days stress (3D); (**C**) five days stress (5D). Physiological effects of drought stress on; (**D**) relative water content (%), (**E**) relative electrolyte leakage (%), and (**F**) MDA content (nmol g^−1^ FW) at C, 3D, and 5D. Bars with two stars (**) are significantly different at *p* ≤ 0.01.

The two inbred lines’ seedlings were subjected to natural drought stress conditions by withholding water for three (3D) and five (5D) days. At well-watered conditions (C), no visible phenotypic differences were observed between the two inbred lines, as they both preserved intact plant architecture (Figure 1A). However, when subjected to 3D and 5D drought stress, the seedlings of LX9801 were more susceptible to drought stress, exhibiting extreme leaf rolling and wilting, compared to the CML69 plants, which showed only minor phenotypic stress (Figure 1B,C). No significant difference was observed in the relative water content (RWC), the relative electrolyte leakage (REL), and leaf malondialdehyde (MDA) content between LX9801 and CML69 at well-watered conditions (Figure 1D–F). The drought-tolerant line (CML69) maintained higher RWC at both 3D and 5D of drought imposition (Figure 1D). However, the REL and MDA content was significantly higher in the susceptible line than the tolerant line at both drought stress conditions (Figure 1E,F, respectively). These results indicate that drought stress might have induced membrane lipid peroxidation in the susceptible line.

### 2.2. RNA Sequencing (RNA-seq) Analysis and Identification of Differentially Expressed Genes

The RNA for the RNA-seq transcriptome analysis was extracted from the leaves of CML69 and LX9801 three-leaf-stage maize seedlings that had been subjected to drought treatment, as stated in the previous section. Three biological replicates were used to represent the control (C), 3D, and 5D samples of the two genotypes, resulting in 18 pairwise comparisons. Eighteen samples were used to make cDNA libraries, subjected to RNA-seq profiling on the Illumina HiSeq™ 2500 platform for deep sequencing. The raw data can be found at https://bigd.big.ac.cn/gsa/ under accession numbers CRA003679 in the Genome Sequence Archive (GSA).

A total of 7.6 billion paired-end reads with a length of 2 × 150 base pairs (bp) were obtained after removing the low-quality sequence and adaptor sequence (Table S1). HISAT2 (Hierarchical indexing for spliced alignment of transcripts) was used to map 1.8 billion clean reads to the maize reference genome B73_v4 (AGPv4, B73 RefGen_v4). The Q30 base percentage, which indicates the overall reproducibility and quality of the assay, was above 91.0%. Moreover, the GC contents of all the reads were above 45%, while the mapping rates of all 18 libraries ranged from 92% to 95%, with the percentages of mapped reads been higher in CML69 than in LX9801 (Table S1).

The degree of expression of each gene was determined using the fragments per kilobase of transcript per million fragments mapped (FPKM) process, and the abundance of gene expression was analyzed using Cufflink’s software [36]. The CummeRbund package was used to estimate the average expression level of both inbred lines genes. The Jensen–Shannon distance dendrogram displayed the similarity among the gene expressions in nine samples of each inbred line and well parallelism among three replicates of each group (Figure S1A). The multidimensional scaling (MDS) plot analysis also showed that each treatment’s replicates clustered together (Figure S1B). Collectively, this analysis highlights the repeatability and the reliability of our results.

**Figure 2:**
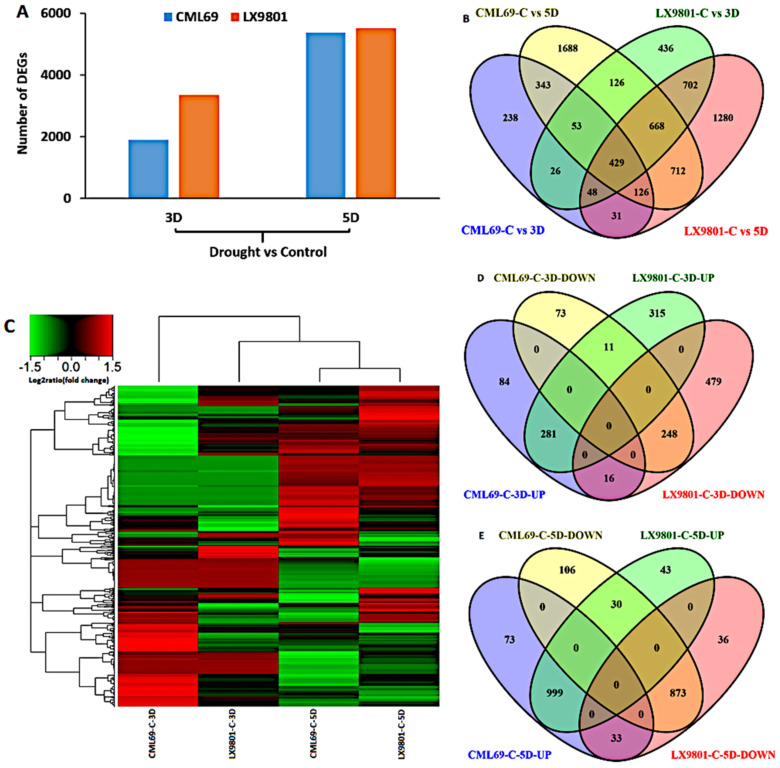
Differentially expressed genes (DEGs) in CML69 and LX9801 during drought conditions. (**A**) Graphic presentation of DEGs in 3D and 5D stress treatment of both CML69 and LX9801. (**B**) Venn diagram showing DEGs’ profile in both inbred lines after drought treatment. (**C**) Heatmap showing the clustering analysis of 2219 common drought-responsive genes. (**D**) Venn diagram showing the comparison of DEGs expressed at 3D drought stress in both inbred lines. (**E**) Venn diagram showing the comparison of DEGs expressed at 5D drought stress in both inbred lines. Drought treatments are labeled as control (C), three days (3D), and five days (5D).

Following 3D and 5D drought stress treatments, gene expression differences in both inbred lines were calculated using the Cuffdiff software package [36]. Generally, a standard foldchange of less or equal to 1 (≥1 or ≤−1), a *p*-value of ≤ 0.05, and a nucleotide length of ≥200 bp were all considered to identify differentially expressed genes (DEGs). A total of 10,084 DEGs were obtained from comparing C versus (vs.) 3D and C vs. 5D of both inbred lines (Figure 2A). A total of 1902 and 3362 DEGs were differentially expressed between C vs. 3D stress of CML69 and LX9801, respectively. Similarly, 5385 and 5512 DEGs were differentially expressed between C vs. 5D drought stress for CML69 and LX9801, respectively (Figure 2A). Among the 10,084 DEGs, the downregulated genes were more than the upregulated genes in both inbred lines for each drought condition (Table 1). Moreover, the numbers of upregulated and downregulated genes were more in the 5D drought imposition of both lines than in 3D of drought imposition (Table 1). In totality, more genes were regulated in the susceptible line than in the tolerant line for both drought stress conditions.

### 2.3. Annotation and Differential Analysis of Differentially Expressed Genes

The maize reference genome B73 RefGen_v4 model was used to annotate 10,084 DEGs, with 6906 (68.5%) DEGs being successfully annotated and 3178 (31.5%) DEGs remaining unannotated. The differential analysis of the 6906 annotated DEGs was carried out as per the inbred line and the magnitude of drought stress. For CML69, 1294 (596 upregulated and 698 downregulated) DEGs were expressed for the 3D drought stress, while 4145 (2154 upregulated and 1991 downregulated) DEGs were expressed for the 5D drought stress (Table 2). Similarly, 2488 (1094 upregulated and 1394 downregulated) and 3996 (2160 upregulated and 1836 downregulated) DEGs were observed in LX9801 at 3D and 5D of drought stresses, respectively (Table 2). We collectively compared 1294, 4145, 2488, and 3996 DEGs to have a complete understanding of our DEGs in response to drought stress.

Based on the overlapping of DEG sets acquired by the above comparison assay, the 6906 annotated DEGs were classified into; genotype-specific responsive genes and common drought-responsive genes (shared by both genotypes) (Figure 2B). There were 2269 genotype-specific DEGs in CML69; 238 and 1688 genes were unique to 3D and 5D, respectively, and 343 genes were common to both 3D and 5D. In the susceptible-line LX9801, 2418 genes were exclusively expressed, with 436 and 1280 genes unique to 3D and 5D, respectively, and 702 genes common to 3D and 5D. In addition, there were 2219 (26, 53, 126, 48, 429, 668, 31, 126, and 712) common drought-responsive genes in both inbred lines at 3D and 5D (Figure 2B).

The 2219 common drought-responsive genes were deemed more critical to the drought stress response of both inbred lines and other maize inbred lines. They indicate the existence of conserved drought-induced regulation pathways between the two genotypes. However, further analysis of these genes showed a disparity in expression patterns between the two inbred lines under 3D and 5D stress (Figure 2C). At 3D stress, CML69 had 713 DEGs (381 upregulated and 332 downregulated), while at 5D stress, 2114 DEGs (1105 upregulated and 1009 downregulated) were expressed (Table 3). Similarly, 1350 (607 upregulated and 743 downregulated) and 2014 DEGs (1072 upregulated and 942 downregulated) were expressed in LX9801 at 3D and 5D drought stress, respectively (Table 3). We also compared all the common drought-responsive DEGs (381, 332, 607, and 743) expressed at 3D stress (Figure 2D) as well as those expressed at 5D stress (1105, 1009, 1072, and 942) (Figure 2E). For CML69, 84 and 73 DEGs were exclusively up-and downregulated, respectively. In comparison, 315 and 479 DEGs were specifically up-and downregulated, respectively, in LX9801 at 3D stress (Figure 2D). A total of 11 DEGs were downregulated in CML69 and upregulated in LX9801 while 16 DEGs were upregulated in CML69 and downregulated in LX9801. Moreover, 281 DEGs were upregulated and 248 DEGs downregulated in both inbred lines at 3D drought stress (Figure 2D). In 5D drought stress, 73 and 106 DEGs were exclusively up-and downregulated, respectively, in CML69, while 43 and 36 DEGs were only up-and downregulated, respectively, in LX9801 (Figure 2E). Furthermore, 30 DEGs were downregulated in CML69 and upregulated in LX9801, while 33 DEGs showed opposite expression patterns in the two inbred lines (Figure 2E).

A total of 284 out of 2613 drought-responsive lncRNAs showed high homology (≥90% identity and ≥80% coverage) to known drought-responsive lncRNAs identified by Zhang et al. [37] (Table S2). Moreover, 144 out of the remaining 2329 drought-responsive lncRNAs showed high homology (≥90% identity and ≥80% coverage) to known lncRNAs identified by Boerner et al. [38] (Table S3). RepeatMasker was used to analyze the repetitive element content of the 2613 drought-responsive lncRNAs. A total of 573 drought-responsive lncRNAs were disguised as repetitive elements, with the most common type of repetitive elements being simple repeats, long terminal repeat retrotransposons (LTRs), and low complexity (Figure S3). Moreover, long interspersed nuclear elements (LINEs) and DNA elements were also observed. These results suggest that drought stress might have regulated multiple putative TE-lncRNAs that might play roles in sensing the physiological or environmental cues affecting the plant.

### 2.4. GO and KEGG Pathway Enrichment Analysis

**Figure 3:**
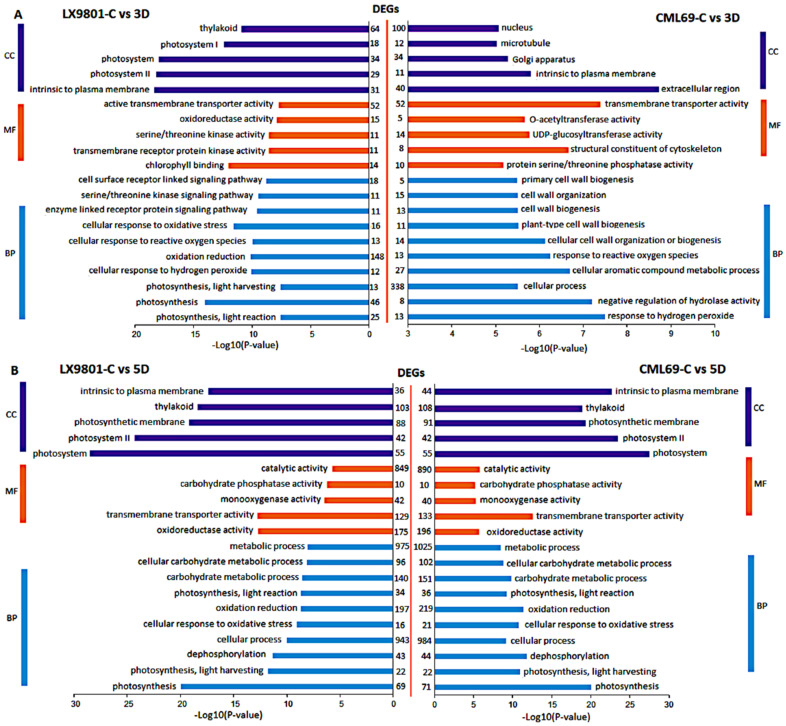
Gene Ontology enrichment analysis of the common drought-responsive genes. (**A**) DEGs expressed at 3D drought stress. (**B**) DEGs expressed at 5D drought stress. The GO terms shown here are the topmost biological process (BP), molecular functions (MF), and cellular component (CC) categories from the tolerant line (CML69) and susceptible line (LX9801). Drought treatments are labeled as control (C), three days (3D), and five days (5D).

The common drought-responsive genes’ roles were elucidated using a gene ontology (GO) enrichment study of the 713, 2114, 1350, and 2014 DEGs. Based on their putative roles, the DEGs were divided into three domains: biological process (BP), molecular function (MF), and cellular component (CC). Photosynthesis was seriously impacted in LX9801 during 3D drought stress, as evidenced by BP GO in photosynthesis. Furthermore, photosynthesis-related CC such as photosystem I and II, as well as thylakoids, were highly enriched in the susceptible line (Figure 3A). Signal transduction was significantly enhanced in LX9801 through BP GO terms of enzyme-linked receptor protein signaling pathway, serine/threonine kinase signaling pathway, and cell surface receptor linked signaling pathway and MF of serine/threonine kinase activity and transmembrane receptor protein kinase activity (Figure 3A). Cell wall modification, on the other hand, was enhanced in CML69 by BP GO in primary cellular cell wall organization or biogenesis, MF of the structural constituent of the cytoskeleton, and CC of microtubule (Figure 3A). Most BP GO terms, photosynthesis, photosynthesis, light reaction, catabolic process, oxidation-reduction, and dephosphorylation, were common in both inbred lines during 5D drought stress (Figure 3B). The majority of MF genes were under catalytic activity, transmembrane transporter, and oxidoreductase activity, while CC related to thylakoid, photosystem I and II, and photosynthetic membrane were enriched in both inbred lines (Figure 3B).

**Figure 4:**
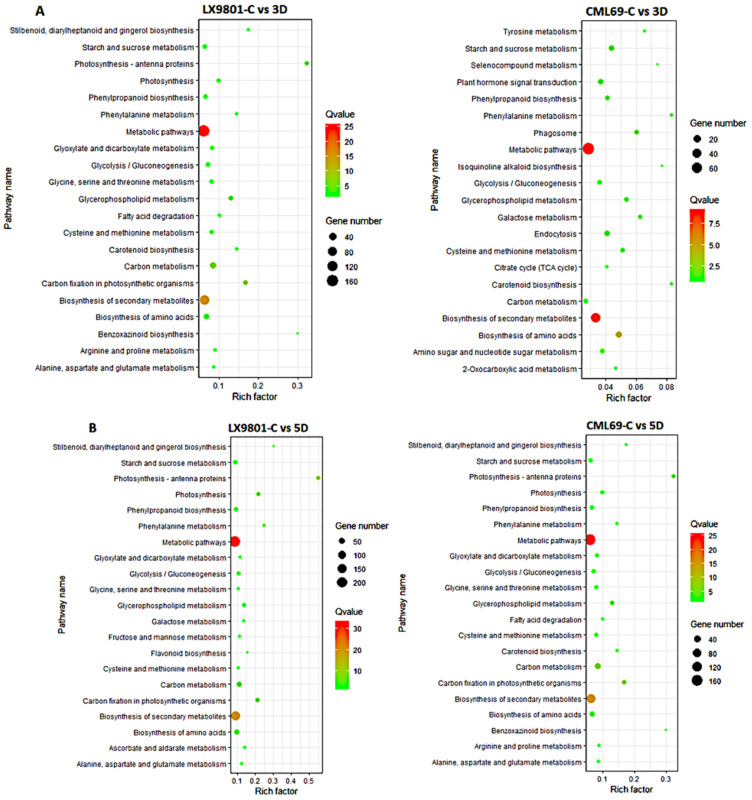
KEGG pathway enrichment analysis of the common drought-responsive genes. (**A**) DEGs expressed at 3D drought stress. (**B**) DEGs expressed at 5D drought stress. The experimental comparisons were based on the hypergeometric test, while the significance of the enrichment of the KEGG pathway is based on the q value (q < 0.05). The “rich factor” shows the DEGs’ ratio to the total gene number in specific pathways. Drought treatments are labeled as control (C), three days (3D), and five days (5D).

**Figure 5:**
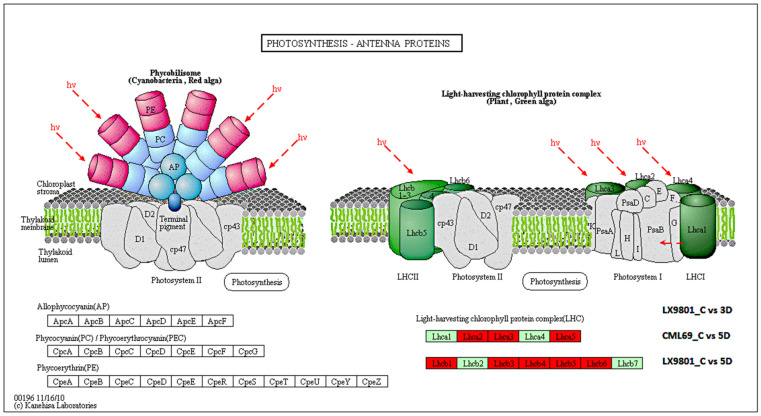
KEGG map of the photosynthetic antenna proteins. It is an analysis of DEGs, comparing drought-treated and control samples in both CML69 and LX9801. Boxes in a red frame indicate that the corresponding DEGs were downregulated in the drought-treated samples, and the boxes with a green frame suggest that the expression levels of the related genes were not changed as determined by our RNA-seq. Drought treatments are labeled as control (C), three days (3D), and five days (5D), and all genes’ abbreviations are defined in Table S9.

**Figure 6:**
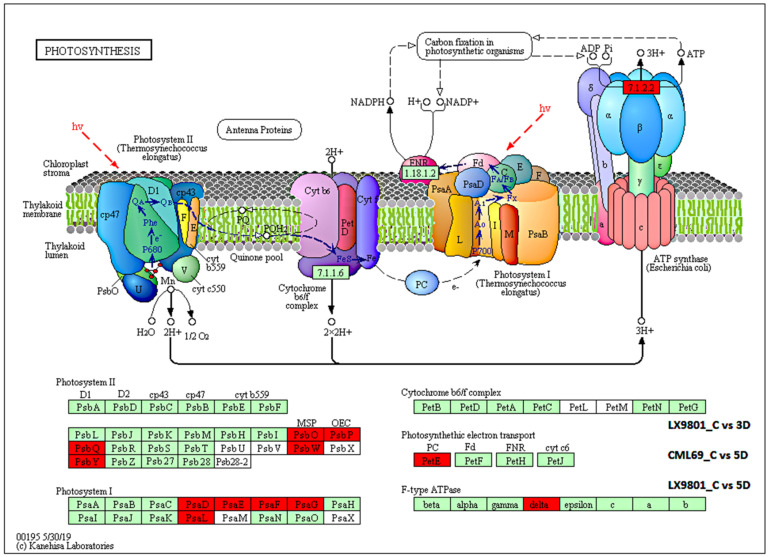
KEGG map of the photosynthesis pathway. It is an analysis of DEGs, comparing drought-treated and control samples in both CML69 and LX9801. Boxes in a red frame indicate that the corresponding DEGs were downregulated in the drought-treated samples, and the boxes with a green frame suggest that the expression levels of the related genes were not changed as determined by our RNA-seq. Drought treatments are labeled as control (C), three days (3D), and five days (5D), and all genes’ abbreviations are defined in Table S9.

The KEGG pathway analysis of the 713, 2114, 1350, and 2014 DEGs showed that during the 3D stress, phagosome, endocytosis, and plant hormone signal transduction were only enriched in CML69. In contrast, photosynthesis and photosynthesis-antenna proteins were only enriched in LX9801 (Figure 4A). Additionally, the functional class of carbohydrate metabolism, lipid metabolism, biosynthesis of other secondary metabolites, and amino acid metabolism were far much enriched in the susceptible line than the tolerant line at 3D drought stress (Figure 4A). In 5D drought stress conditions, most pathways such as photosynthesis, photosynthesis-antenna proteins, starch, and sucrose were enriched in both inbred lines though the enrichment was more in the susceptible line (Figure 4B). Nevertheless, pathways such as starch and sucrose metabolism, amino sugar and nucleotide sugar metabolism, phenylalanine, tyrosine and tryptophan biosynthesis, and glycolysis/gluconeogenesis were enriched in all the drought conditions of both inbred lines during carbon fixation in photosynthetic organisms, glyoxylate and dicarboxylate metabolism, and photosynthesis. Antenna proteins were enriched in all drought conditions except the 3D drought stress of CML69 (Figure 5 and Figure 6).

### 2.5. Effects of Drought Stress on the Drought-Tolerant Line

The 84 DEGs upregulated exclusively in CML69 under 3D stress were significantly enriched in the GO terms of cellular carbohydrate metabolic process, cell cycle, and macromolecule localization (Table S4). Moreover, KEGG pathway analysis of the same DEGs highlighted enrichment of glycolysis/gluconeogenesis, isoquinoline alkaloid biosynthesis, endocytosis, and amino sugar and nucleotide sugar metabolism pathways (Table S4). The expression of genes involved in cell division and growth (ATK5, CDKG1, and CDKC2), as well as cytoskeleton-related genes (2TUB8), indicates that they may play a role in modulating CML69 growth under 3D stress. Cell wall-related genes (3CESA, SUS3, RPG, TBL, UXS, and UGE), lignin biosynthetic genes (CAD, PRX52, LAC17, MYB63, and MYB4), suberin biosynthetic genes (GPAT5 and HXXXD-type acyl-transferase family protein), and cuticular wax biosynthetic genes (CER3, KCS11) were all upregulated indicating the role of the cell wall in providing mechanical strength to CML69 during 3D drought stress condition thereby withstanding the turgor pressure (Table S4). The secondary metabolites regulated genes (TAT3, TAT7, CYP75B1, and CHIL), transport genes (SWEET2, aquaporin (TIP3), and glycolysis/gluconeogenesis pathway genes (TPI, ENO1, and FBA2), and DREB1A encoding gene were all enhanced in CML69 at 3D drought conditions (Table S4). In addition, endocytosis-related genes (ARFA1F, RABA4a), late embryogenesis abundant (LEA), NPF3.1, and ESK1 genes, which facilitate the adaptation of the plant to water deficit, were also enhanced in CML69 at 3D stress condition (Table S4).

The 73 exclusively downregulated DEGs were significantly enriched in the GO terms of the cellular catabolic process, cellular carbohydrate metabolic process, proteolysis, and transmembrane transport (Table S5). The KEGG pathways analysis of the same DEGS highlighted the enrichment of plant hormone signal transduction and starch and sucrose metabolic pathways (Table S5). Downregulation of transmembrane transporter-related genes (ABC, SWEET17, DTX33, CAT8, BASS1, GPT2, and MTP11) during drought stress could be a major factor in nutrient redistribution. Phytohormones related genes such as auxin (IAA16, TCH4, and SAUR59) and abscisic acid (PYR1) suggest their essential roles in coordinating different signal transduction pathways at 3D stress conditions. Moreover, downregulation of sugar-related genes (TPS1, TPS6, and SUS4) might suggest the complexity of osmotic adjustment during drought stress conditions (Table S5). Interestingly, cytoskeleton (ACT7, ACT11) and cell wall (PRCW, BG3, and GATLW) related genes were significantly expressed in the 16 DEGs that were upregulated and downregulated in CML69 and LX9801, respectively, at 3D drought stress (Table 4). Similarly, expression of genes encoding phosphate transporters (PHT1 and PHT7), vitamin B6 (PEPC1), flowering (FPF1), seed maturation (AATP1), ABA biosynthesis (ASR3), Aspartic protease (SAP2), and CYP450 protein (CYP72A15) were all enhanced in the tolerant line and repressed in susceptible line at 3D drought stress (Table 4). These 16 DEGs may be the most critical factors in CML69’s drought stress tolerance.

The 73 DEGs exclusively upregulated in CML69 at 5D stress were significantly enriched into GO terms of localization, catabolic process, oxidation-reduction, and KEGG pathways of ABC transporters, phagosome, endocytosis, and metabolic pathways (Table S6). Transport-related genes such as 4PHT, 2ABC, TOM, and UMAMIT19 were all induced by drought stress suggesting their roles in drought stress tolerance of CML69. The auxin transporter gene (PIN1) was also enhanced by drought, implying that the auxin hormone’s concentration was altered. Other genes enriched in CML69 at 5D drought stress include; CCD7, whose activity on β-carotene affect the content of the epoxy carotenoids, the precursors to ABA, A/N-INVI gene, which breaks down sucrose, two secreted plant proteases (SAP2) genes that serve as a front line of immunity through inhibiting bacterial growth, two chitinase encoding genes (CHIA, CHIB) that target fungal pathogens by catalyzing the degradation of the fungal cell wall, serine/threonine protein kinase (ATM) gene that results to stress-induced programmed cell death, and clathrin heavy chain genes (2CHC), which re-establishes the cellular osmotic balance by the compartmentalization of endomembranes or membrane proteins under water stress (Table S6). Moreover, the 106 DEGs that were exclusively downregulated in CML69 at 5D stress were enriched into GO terms of oxidation-reduction, catabolic process, and response to inorganic substance and KEGG pathways of phenylpropanoid biosynthesis, peroxisome (Table S7). Surprisingly, Reactive oxygen species (ROS) scavengers such as CAT, PRX, PRXQ, and SOD were all downregulated in CML69 suggesting the complexity of the antioxidant process during drought stress. Two CYP450 encoding genes (CYP93D1 and CYP94C1), and PAO1, which function in polyamine catabolism, were also downregulated (Table S7).

As shown in Figure 2E, 33 DEGs were upregulated in CML69 and downregulated in LX9801 at 5D stress (Table 5). These genes might be crucial to the difference in drought stress response between the two inbred lines at 5D. Among them were three acyl lipid metabolism (ALP) encoding genes (ASFT, 4CL, and T5PTASE9), which are vital for suberin synthesis. Additionally, the expression of other cell wall-related genes such as GH, and EXPB4, which contribute to the modulation of cell wall architecture, were also enhanced (Table 5). Glycosyltransferases (GTs) encoding genes (GALT29A, GALT61, GALT, and UGT85A2), transport-related genes (AAP6, AMT1), acid phosphatases genes (PAP3, *Zm00001d025724*), protein kinase genes (PPCK, CIPK), and AAA-ATPase (2 AATP1) were all enhanced implying their essential roles in drought stress tolerance of CML69 (Table 5).

### 2.6. Effects of Drought Stress on the Drought-Susceptible Line

Transcriptome analysis revealed that LX9801 was affected by drought stress more than CML69 at 3D of drought conditions. Two-fold of the number of DEGs expressed in CML69 was observed in LX9801 (Table 1). At 3D drought stress, 315 DEGs were exclusively upregulated in LX9801, while no changes were observed in CML69 (Figure 2D). The GO analysis of the 315 upregulated DEGs highlighted the enrichment of GO terms of catabolic process, oxidation-reduction, carbohydrate catabolic process, glycerolipid metabolic process, cellular amino acid, and derivative metabolic process (Table S8). Moreover, the KEGG analysis of these 315 DEGs highlighted the significant enrichment of glycerophospholipid metabolism, Benzoxazinoid biosynthesis, fatty acid degradation, alanine, aspartate, and glutamate metabolism, pyruvate metabolism, and cutin, suberin, and wax biosynthesis pathways (Table S8). Carbohydrates related genes such as the SPS (*Zm00001d050125*) gene, which is involved in the synthesis of sucrose in the cytosol, and A/N-INVI (*Zm00001d004804* and *Zm00001d051666*) genes, which irreversibly cleave sucrose into fructose and glucose, were enhanced at 3D drought stress. In addition, the FRK gene expression that plays a role in the phosphorylation of fructose to fructose-6-phosphate was enhanced. Fructose-6-phosphate can then be metabolized via glycolysis or used in sucrose and starch biosynthesis. Moreover, an SS2 and a BAM gene, which functions in starch synthesis and degradation, respectively, were also upregulated in LX9801. These results suggest that starch biosynthesis and degradation, as well as sucrose accumulation, were all enhanced in LX9801 with no comparable changes in CML69 at 3D of drought stress. Cell wall degradation enzymes such as 2MAN, GH, UGE, and GBA2 were all enhanced by drought stress in LX9801. Such enzymes are believed to weaken the cell wall barrier, thereby facilitating the pathogenic attack. These might be the reason why benzoxazinoid biosynthesis encoding genes (bx1, bx2, and bx3), which are triggered by pest attacks, were enhanced in LX9801 (Table S8). Glycerophospholipid encoding genes (2PLD, 2DGK, 2PGP, PSS, GPDHC, CCT, and PECT), and amino acid encoding genes (ASP, AGT, TAT, ADC, GAD, PGDH, SAMDC, and ASN), cuticular wax (CLO4, CER1), and gibberellins (GA3ox, GA2ox) were all enhanced in LX9801 at 3D drought stress (Table S8).

The GO analysis of the 479 DEGs that were exclusively downregulated in LX9801 highlighted the enrichment of the GO term of photosynthesis, photosynthesis light reaction, photosynthesis light-harvesting, signaling pathway, oxidation-reduction, catabolic process, dephosphorylation as well as KEGG pathways of energy metabolism (photosynthesis-antenna proteins, photosynthesis, carbon fixation in photosynthetic organisms), carbohydrates metabolism (glycolysis/gluconeogenesis, pyruvate, glyoxylate and dicarboxylate, galactose metabolism, and starch and sucrose metabolism) (Table S9). The 3D drought stress significantly reduced LX9801’s photosynthesis capability, contributing considerably to the “source” for plant growth and development. The 13 genes (lhca2, lhca3, lhca5, 6lhcb1, lhcb3, lhcb4, lhcb5, and lhcb6), which encodes subunits of the light-harvesting chlorophyll protein (LHC) complex responsible for absorbing light and passing it to the light reaction center of the corresponding photosystem were downregulated in LX9801 (Table S9, and Figure 5). Moreover, in the photosynthesis pathway, 10 genes (2PsbO, 3PsbP, 3PsbQ, PsbW, and PsbY) encoding protein subunits of photosystem II reaction center pigment-protein complex (PSII-RC), which function as the light reaction center, were all downregulated in LX9801. Moreover, genes encoding F-type ATPase (*Zm00001d018069*), photosynthetic electron transport (PetE), and photosystem I reaction center (PsaD, PsaE, PsaF, PsaG, and PsaL) were all downregulated in LX9801 at 3D drought condition (Table S9, and Figure 6). The expression profiles of photosynthesis and photosynthesis. Antenna proteins suggest that photosynthesis was diminished in LX9801 as early as 3D of drought stress but was not affected in CML69. Carbohydrate metabolism pathways were significantly reduced in LX9801 at 3D drought stress. Genes encoding carbon fixation in photosynthetic organisms such as FBA, 2FBP, GAPB, MDH, 2PGK, PPDK, 2PRK, 2RBCS, SBPASE, TIM, and TKL1 were all downregulated in LX9801 while no change was observed in CML69 at 3D drought stress (Table S9). These results imply that carbon fixation might have been impaired due to abolished photosynthesis in LX9801 as early as 3D drought conditions.

The activation and modulation of a significant number of drought-responsive genes are related to a signaling pathway mediated by plasma membrane receptors via stress perception. Stress signal then activates several cascades such as phosphorylation and dephosphorylation mediated by several protein kinases and phosphatases, which then activate several downstream genes. Herein, under the signaling pathway and protein modification process, protein kinase encoding genes such as 2WAK, 4LecRK, and 3PK were all downregulated by drought stress in LX9801 (Table S9). Different kinds of protein kinase such as calmodulin-domain protein kinase, CBL-interacting protein kinases, cysteine-rich RLK, lectin protein kinase family protein, leucine-rich repeat protein kinase, and protein kinase superfamily protein were all downregulated. Under the macromolecule metabolic process, various transcription factors such as AP2, ARF, 6bHLH, CO-like, GATA, HSP, 4ERF, FAR1, 2MYB, NAC, 4WRKY, 2TCP, HD-ZIP, NF-YA, and MYB_related were all downregulated by drought stress in LX9801 (Table S9). This highlights their role in regulating downstream genes during drought stress. As shown in Figure 2D, 11 DEGs were downregulated in CML69 and upregulated in LX9801 at 3D drought stress (Table 6). Transmembrane transport encoding genes such as NPF, OCT, and PUMP, which are involved in the transportation of various components involved in osmotic regulation and growth and development of plants during drought stress, were enhanced in LX9801 by drought stress. The expression of MT2 and GSTU encoding genes involved in detoxification processes was enhanced in LX9801 (Table 6).

The GO analysis of the 43 DEGs exclusively upregulated in LX9801 at 5D drought stress highlighted enrichment of the GO terms of microtubule-based process, RNA biosynthetic process as well as KEGG pathway enrichment of endocytosis, Phagosome, biosynthesis of amino acids, and phenylalanine metabolism (Table S10). Microtubule encoding genes such as ARFA1F, TUA6, CH, TUB6, and TUB8 were all enhanced at 5D stress in LX9801 (Table S10). RNA biosynthetic process GO term highlighted that transcription factors (TFs) such as NAC041, ERF11, RAP2.6, ERF53, and FITNESS were all upregulated (Table S10). These TFs might have regulated the downstream genes responsible for the differences in the drought stress response of the two inbred lines. Expression of endocytosis-related genes (ARF, RAB) involved in various regulatory processes of plant development and stress response was also enhanced (Table S10). Moreover, the GO analysis of the 36 downregulated DEGs in LX9801 at 5D drought stress highlighted GO terms of response to the bacterium, phenylpropanoid metabolic process, defense response, and response to abiotic stimulus, as well as KEGG pathway enrichment of glycerophospholipid and galactose metabolism (Table S11). Genes encoding ESE3, CAT1, AAP6, UGT, and GATL were all downregulated at 5D drought stress in LX9801 (Table S11). A total of 30 DEGs were upregulated in LX9801 and downregulated in CML69 at 5D stress (Table 7). Benzoxazinoid biosynthesis genes (bx1, bx2, and bx3) are involved in defense response against various lepidopteran pest attacks, and their induced expression in LX9801 might suggest pathogenic microorganisms attack LX9801 during drought stress. The uptake of ions, water, nitrogen metabolism, and nutrient redistribution were affected differently at 5D drought stress between the two inbred lines as reflected by the opposite expression of nitrogen metabolism (GSR, NIA), nitrate transporter (NPF7.3 and NPF7.3), LHT, GPT2, and TIP2 encoding genes. The TFs of the family MYB, GRF2, GLK44, and KDR TFs were also enhanced by a 5D drought stress suggesting their crucial role in the drought stress response of LX9801 (Table 7).

### 2.7. Validation of DEGs by Quantitative Real-Time PCR (qRT-PCR)

**Figure 7:**
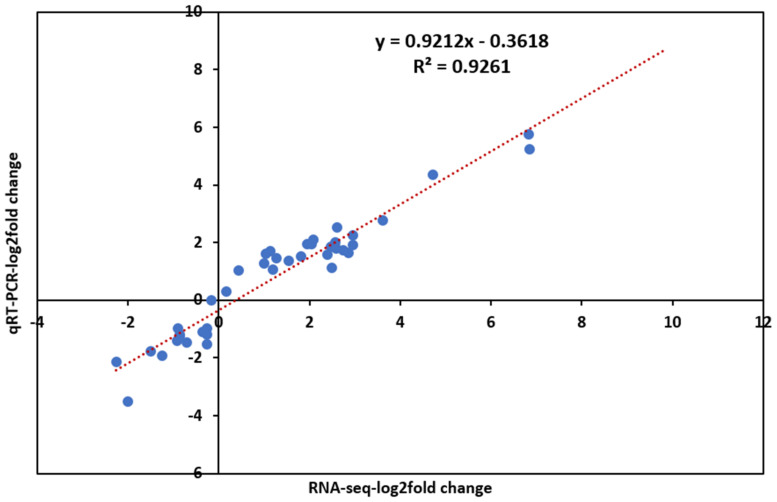
Correlation analysis between RNA-seq and qRT-PCR methods. Log2fold values of RNA-seq data (x-axis) are plotted against log2fold values of qRT-PCR (y-axis) data.

To confirm the reliability and validity of the RNA-seq results in maize seedlings, 10 DEGs were selected at random for quantitative real-time PCR (qRT-PCR) analysis in 3D and 5D stress. The ratio of the expression levels between control and treatment was calculated and compared with the foldchange obtained from RNA-seq. A high significant correlation (R2 = 0.9261) between RNA-seq and qRT-PCR data was observed (Figure 7), which confirmed the authenticity of the DEGs in this study.

## 3. Discussion

The physiological indices highlighted that the tolerant and susceptible inbred lines responded differently at drought stress conditions. By taking into account the rolling and drying of the leaves, the tolerant line exhibited a reduced degree of rolling and drying of the leaf than the susceptible line (Figure 1). Delayed leaf rolling in plants has been postulated as a drought escape mechanism through the adjustment of their leaf water potential, thereby absorbing soil water more efficiently [39]. The tolerant-line seedlings exhibited higher RWC than susceptible-line seedlings at both 3D and 5D drought conditions (Figure 1D). High RWC might help the tolerant line to perform various physio-biochemical processes more efficiently under drought stress than the susceptible line. The rate of REL represents the degree of damage to the plant cell membrane under osmotic stress, while the content of MDA is the final decomposition product of membrane lipid peroxidation. Thus, the lower REL and MDA content in the tolerant line (Figure 1E,F) could indicate a higher cell membrane stability index under drought stress conditions. Higher RWC and cell membrane stability help the plant to endure moisture deficit under drought stress conditions [40,41]. Interestingly, our RNA-seq findings were in agreement with our physiological analyses results that the two maize inbred lines responded quite differently to the drought stress. The tolerant line had comparatively fewer DEGs than the susceptible line at both 3D and 5D drought stress conditions (Figure 2, Table 1). Higher RWC, lower electrolyte leakage, and lower MDA content in the tolerant line might imply that there was relatively lower stress at a cellular level and thus a more limited transcriptomic response compared to the susceptible line. Previous transcriptomic studies in maize [42] and rice [43] observed a similar trend where the tolerant line had fewer DEGs expressed than the susceptible line under stress conditions.

The cell wall is a dynamic polysaccharide network that offers plant stability and protection under drought stress conditions. Stress-induced perturbations at the cell wall alter the synthesis of cellulose and the arrangement of microtubules to respond to environmental stresses [44]. The cell wall metabolism-related genes such as CESA (*Zm00001d005775*, *Zm00001d020531*, and *Zm00001d032776*), RPG (*Zm00001d022085*), TBL (*Zm00001d048608*), SUS (*Zm00001d029087*), GATL2 (*Zm00001d033385)*, GALT (*Zm00001d003091*, *Zm00001d011838*, and *Zm00001d024687*), PRCW (*Zm00001d024027*), alpha-mannosidase (*Zm00001d030285*), and EXPB4 (*Zm00001d017494*) were all upregulated in the tolerant line at 3D drought stress (Table S4 and Table 4). TBL participates in secondary wall cellulose synthesis, while enhanced CESA and SUS suggest a potentially higher cellulose biosynthesis in the tolerant line. Cellulose plays a key role in maintaining the integrity of the cell wall and cell turgor pressure, thus allowing continuous cell growth under low water potential [45]. A previous study by Zheng et al. [46] observed an increase in cellulose levels in cotton under drought stress. GATL genes are involved in the pectin and/or xylan biosynthesis of the cell wall [47], while PRCW covalently binds to pectin or hemicellulose during abiotic stress, thus helping to strengthen the cell wall [48]. Alpha-mannosidase is significant in glycan maturation, which is important for sufficient cell wall formation under drought stress, while EXPB has become widely acknowledged as key regulators of cell wall extension and modification, particularly during water stress conditions [49]. Lignin biosynthesis genes such as CAD (*Zm00001d024314*), PRX52 (*Zm00001d053554*), LAC17 (*Zm00001d042906*), MYB63 (*Zm00001d002476*), MYB4 (*Zm00001d041853*), WAT1 (*Zm00001d035717*), and 4CL (*Zm00001d032103*) were also enhanced in the tolerant line at 3D and 5D drought stress (Table S4 and Table 5). The 4CL gene catalyzes the metabolic pathway related to lignin [50], which is a fundamental component of the plant’s secondary cell wall. Lignin content and composition have previously been reported to change during drought stress conditions [51]. Moreover, suberin biosynthetic genes such as HXXXD-type acyl-transferase (*Zm00001d037619*, *Zm00001d046455*), GPAT5 (*Zm00001d038366*), ASFT (*Zm00001d007606*), and cuticular wax biosynthetic genes such as CER3 (*Zm00001d046865*), KCS11 (*Zm00001d039094*) were all enhanced by drought stress in the tolerant line at 3D and 5D drought stress (Table S4 and Table 5). Suberin is a lipophilic cell wall barrier that regulates fluxes of water and nutrients and restricts pathogen infection during abiotic stress [52,53]. Accumulation of cuticular waxes has also been reported to contribute to drought resistance [54]. Taken together, the upregulation of cell wall-related genes suggests that cell wall modification may be a protective strategy of the tolerant line against drought stress, and thus, it is an essential adaptive response to drought stress in maize seedling.

The plant cytoskeleton (microtubules and actin filaments) incorporates vibrant elements that are essential to the stress resistance and tolerance of plants [55]. In this study, TUB (*Zm00001d013612*, *Zm00001d015348*), ATK (*Zm00001d002186*), CDKG1 (*Zm00001d041826*), CDKC2 (*Zm00001d011967*), and ACT (*Zm00001d008746*, and *Zm00001d013410*) genes were enhanced by drought stress in the tolerant line (Table S4). ATK5 is a molecular motor protein that is essential in mitosis during cell division, while CDKC2 affects both cell division and plant response to drought stress by changing the stomatal density [56]. CDKG1 modulates the function of SINA2 ubiquitin ligase to control its effect on ABA and osmotic stress responses in plants [57]. This implies the role of cell cycle genes in modifying growth patterns of the tolerant line by coordinating the rate of cell proliferation and expansion. Alteration of plant growth by drought stress consequently changes the organization and dynamics of the cell wall and cytoskeleton-related proteins [58]. The increase in the abundance of cytoskeleton-related genes in the tolerant line might have affected the polymerization and alignment of the cytoskeleton, which further affects cell stability and plant resistance to drought stress. Our findings are in agreement with Jiaowen et al. [59] study, which reported the involvement of the cytoskeleton-related protein in drought tolerance of foxtail millet.

Photosynthesis-related processes are the most susceptible to drought stress owing to stomatal closure, which reduces the intake of CO_2,_ thereby affecting the rate of photosynthesis and consequently affecting plant growth and yield [21,30,60]. Thus, maintenance of photosynthetic rates under drought stress is essential for drought tolerance in plants [61]. In this study, 13 DEGs encoding LHCA/B were all downregulated in the susceptible line, with no changes observed in the tolerant line at 3D drought stress (Table S9). Antenna protein present in LHC protein complexes acts as a peripheral antenna system, allowing more efficient absorption of light energy. Therefore, the downregulation of LHC implies that the absorption of light energy might have been limited in the susceptible line at 3D drought stress. Moreover, 17 DEGs encoding different categories of photosynthesis such as F-type ATPase, photosynthetic electron transport, PS1, and PS11 were all downregulated in the susceptible line with no changes observed in the tolerant line at 3D drought stress (Table S9). This implies that the photosynthetic electron transport process, the synthesis of ATP, and the binding stability of PS1 were inhibited in the susceptible line at 3D drought stress. MYB60 encoding gene (*Zm00001d023282*) has been reported to act as a stomatal movement regulator [62]. Downregulation of this gene in the tolerant line might suggest a decrease in the stomatal aperture that helps to limit water loss during drought. Collectively, our results indicate that the photosynthesis process was severely affected in the susceptible line as reflected by the significantly reduced transcript abundance of a broad spectrum of required genes resulting in a lower photosynthetic rate.

Carbohydrate metabolism plays a significant role in maintaining plant normal growth and development under drought conditions [30]. Previously, the expression pattern of genes involved in carbohydrate metabolism induced changes in carbohydrate content in various plants under drought stress conditions [63]. Multiple metabolic pathways encoding genes such as FBA2 (*Zm00001d053015*), FBP (*Zm00001d028562* and *Zm00001d042727*), GAPB (*Zm00001d027488*), MDH (*Zm00001d022229*), PGK2 (*Zm00001d010672*, and *Zm00001d038579*), PPD (*Zm00001d010321*), PRK (*Zm00001d002454*, and *Zm00001d017711*), TIM (*Zm00001d021310*), TKL (*Zm00001d045451*), and RBCS (*Zm00001d052595*, and *Zm00001d004894*) were all downregulated in the susceptible line with no observed changes in the tolerant line at 3D drought stress (Table S9). Ribulose-bisphosphate carboxylase (RBCS), is a key gene in carbon fixation, which catalyzes the incorporation of carbon dioxide (CO_2_) into ribulose 1, 5-bisphosphate, thereby playing an important role in CO_2_ assimilation. Downregulation of RBCS genes leads to suppression of CO_2_ assimilation, thereby lowering the photosynthetic rate in the susceptible line. In addition, genes related to SS2 (*Zm00001d037234*), BAM (*Zm00001d027619*), SPS (*Zm00001d050125*), A/N-INVI (*Zm00001d004804* and *Zm00001d051666*) were upregulated in the susceptible line with no observed change in tolerant line at 3D drought stress (Table S8). SS2 and BAM genes function in starch synthesis and degradation, respectively. SPS gene is involved in the synthesis of sucrose in the cytosol, while A/N-INVI genes irreversibly cleave sucrose into fructose and glucose. Thus, starch synthesis and degradation, which contributes to carbohydrate reserves, were enhanced in the susceptible line. Moreover, a GPT gene (*Zm00001d021653*) was upregulated in the susceptible line and downregulated in the tolerant line at 5D drought stress (Table 7). GPT is the preferred substrate for starch synthesis in guard cells, indicating that metabolites were diverted from soluble sugars to starch resulting in reduced osmotic potential in the susceptible line at drought conditions [64]. Collectively, our results suggest that drought stress-regulated diverse sugar-related genes in the susceptible line. This high sugar demand might be due to lower carbohydrate assimilation as a result of abolished photosynthesis in the susceptible line.

Drought tolerance is an intensive phenomenon that requires a substantial amount of energy to cope with it. Inorganic pyrophosphatases generate the thermodynamic driving force (ATP) for some cellular biosynthetic reactions by catalyzing the hydrolysis of inorganic pyrophosphate to inorganic orthophosphate. An inorganic pyrophosphatase (*Zm00001d011734*) encoding gene was upregulated in the tolerant line and downregulated in the susceptible line at both 3D and 5D drought stress (Table 4 and Table 5). Moreover, glycolysis/gluconeogenesis-related genes such as TPI (*Zm00001d012407*), ENO (*Zm00001d020309*), and FBA (*Zm00001d042279*) were all upregulated in the tolerant line with no changes observed in the susceptible line at 3D drought stress (Table S4). Moreover, a phosphofructokinase encoding gene (*Zm00001d006857*) was downregulated in the tolerant line and upregulated in the susceptible line (Table 7). TPI catalyzes the reversible interconversion of dihydroxyacetone phosphate and D-glyceraldehyde 3-phosphate for efficient energy production in glycolysis [65]. Enolase catalyzes the dehydration of 2-phosphoglycerate to phosphoenolpyruvate, which has been reported in response to salt and drought stress [66]. Enhanced enolase translates into an improved glycolysis pathway leading to the accumulation of acetyl-CoA in the Krebs cycle resulting in a large amount of ATP for drought tolerance. The upregulation of glycolysis related enzymes were previously reported in drought-tolerant soybean [67]. Reduced expression of phosphofructokinase 4 gene favors the conservation of energy resources, thereby contributing to drought tolerance [33]. This coordinated induction might be crucial for activating the entire energy-producing pathway in the tolerant line to sustain major physiological activities and inhibit drought stress damage.

Plant aquaporins (AQPs) play a significant role in drought tolerance by facilitating water and small solute transport across the cell membrane and thus regulate plant growth and development [68]. A TIP3 gene (*Zm00001d048520*) was upregulated in the tolerant line with no observation in the susceptible line at 3D drought stress (Table S4). In addition, a TIP2 encoding gene (*Zm00001d051362*) was downregulated in the tolerant line and upregulated in the susceptible line at 5D drought stress (Table 7). The upregulation of TIP3 might have facilitated water and solute transport across the membranes of the tolerant line at 3D stress, while downregulation of TIP2 at 5D might be a way to minimize water flow through cell membranes and uphold leaf turgor, thereby helping the tolerant-line seedlings from being affected by drought stress. Amino acid transporters are highly regulated by abiotic stresses [69]. Genes encoding CAT1 (*Zm00001d033241*) and AAP6 (*Zm00001d018751*) were upregulated at 3D and 5D drought stress in the tolerant line but downregulated in the susceptible line (Table 4 and Table 7). AAP6 transports different amino acids such as proline (osmoprotectants), thus helping the tolerant line to survive at extreme osmotic stress [70]. Maintaining the homeostasis of inorganic phosphate (Pi) is essential for the growth and yield of plants. Upregulation of genes encoding acid phosphatase (*Zm00001d025724* and *Zm00001d028367*) and SPX3 (*Zm00001d044541*) maintained a certain level of inorganic phosphate in the tolerant line, which can be co-transported with H^+^ along a gradient of proton motive force. Similarly, the upregulation of PHT7 (*Zm00001d031875*) and PHT1 (*Zm00001d032850*) in the tolerant line (Table 4) implies an influx of Pi and translocation of the same in the tolerant line. The expression of PHT genes has been reported to play a fundamental role in osmotic adjustments during plant responses to water deficit. Moreover, ammonium (NH_4_^+^) and nitrate (NO_3_^−^) ions are mediated by ammonium (AMTs) and nitrate (NRTs) transporters, respectively [71]. Nitrate reductase (NR) catalyzes the conversion of nitrate to ammonium, where the latter is assimilated by glutamine synthetase (GSR) into amino acids [71]. In the current study, genes encoding NRT1.1 (*Zm00001d043374*), NRT1.5 (*Zm00001d017666*), NR2 (*Zm00001d018206*), and GSR1 (*Zm00001d033747*) were downregulated in the tolerant line but upregulated in the susceptible line at 5D drought stress condition (Table 7). NRT1.1 is a dual transporter required for the development of young organs, stomatal opening, and contributes to drought susceptibility [72]. Downregulation of NRT1.1 might participate in altering root morphology that may in turn help the tolerant line to withstand stress conditions, while repressed expression of NRT1.5 and NR2 contributes essentially to drought stress tolerance through reallocating nitrate to plant roots. Reduced expression of the GS gene may slow down the process of ammonia assimilation and thus affects nitrogen metabolism. Moreover, GS is an ATP-dependent enzyme that fixes the ammonium into glutamate to form glutamine. On the other hand, the upregulation of the AMT1; 2 (*Zm00001d017249*) gene in the tolerant line (Table 5) implies that NH_4_^+^ has a vital role in conferring drought tolerance in the tolerant line. The absorption of ammonium ion in plants is energetically less expensive as plants do not have to use additional energy for reducing nitrate into ammonium. Previous studies have reported that NH_4_^+^ reduces the detrimental effects of drought stress [73]. Collectively, multiple transport-related genes were key drought enhancers in the tolerant line through maintaining water, ions, and nutrients homeostasis.

Late embryogenesis abundant (LEA) proteins are highly hydrophilic and thermally stable, with a major role in abiotic stress tolerance in plants [74]. The LEA encoding gene (*Zm00001d008850*) was upregulated in the tolerant line with no observation in the susceptible line at 3D drought stress (Table S4). The upregulation of LEA protein might have protected the tolerant-line cellular structures from injuries by maintaining orderly structures within the cell. Vitamin B6 can quench oxygen singlets, superoxide anion radicals and regulate cell signaling molecules and ion channels associated with cell membranes under drought conditions [75]. Genes encoding PEPC1 (*Zm00001d016301*) and PPCK (*Zm00001d017270*) upregulated at 3D and 5D drought stress in the tolerant line but downregulated in the susceptible line (Table 4 and Table 7). PEPC1 catalyzes pyridoxal 5-phosphate (PLP) to pyridoxal, an active form of vitamin B6. A study by Bin Dong et al. [76] reported that vitamin B6 played an antioxidant role in tea oil camellia under drought stress. Enhanced PPCK increases PEPC activity upon drought stress, thereby improving carbon metabolism during periods of reduced stomatal conductance by reassimilating respired CO_2_ and/or increasing rates of CO_2_ fixation at night when stomata are open [77]. Moreover, PEPC has also been reported to support the biosynthesis of biocompatible osmolytes such as proline that plays significant roles during drought stress conditions [78].

Plants express defense-related genes such as pathogenesis-related proteins (PR) during abiotic stress environments. In this study, defense-related genes such as WAKL (*Zm00001d050164*), SCPL22 (*Zm00001d037797*), and PR4 (*Zm00001d048947*) were all upregulated in the tolerant line at 3D and 5D drought stress but downregulated in the susceptible line at 5D drought stress (Table 5). Over-expression of the PR4 gene in transgenic rice enhanced drought tolerance at both seedling and reproductive stages [79], while WAKL genes protect the plant from pathogenic infections. A BG3 gene (*Zm00001d042143*) catalyzes the hydrolytic cleavage of beta-1, 3-glucans, thereby inhibiting the growth of pathogens was enhanced in the tolerant line but downregulated in the susceptible line during 3D drought stress (Table 4). A previous study by Akiyama et al. [80] reported that rice BG3 (*OsGLN1*) was upregulated by drought stress. Chitinase encoding genes CHIA (*Zm00001d037656*), CHIB (*Zm00001d036370*) were downregulated at 3D drought stress in the susceptible line with no observed change in the tolerant line but upregulated at 5D drought stress in the tolerant line with no observed change in the susceptible line (Table S6). Enhanced activities of BG3 and CHI were reported in white clover leaves under drought stress [81]. Benzoxazinoid biosynthesis genes such as BX1 (*Zm00001d048709*), BX2 (*Zm00001d048710*), and BX3 (*Zm00001d048702*) were upregulated at 3D and 5D drought stress in the susceptible line but downregulated at 5D drought stress in the tolerant line (Table 7). BX1–3 are involved in biochemical defense against a variety of biotic stresses, including insect herbivores, microbial pathogens, and competing plant species. Other defense-related genes such as MT2A (*Zm00001d039859*), GST (*Zm00001d043795*), UGT74F1 (*Zm00001d021168*), and Saf1 (*Zm00001d028693*) were all upregulated in susceptible line and downregulated in tolerant line at 5D drought stress (Table 6). Overall, our results highlight diverse defense mechanisms in both inbred lines and the existence of overlaps in the adaptive mechanisms between biotic and abiotic stresses.

Plants tend to cope with drought stress through the adjustment of their flowering time to the most appropriate moment of the vegetative season. The flowering time determines the ASI of maize through the regulation of flowering time genes. Herein, genes encoding for FPF1 (*Zm00001d004203*) and AATP1 (*Zm00001d007773*, *Zm00001d047436*) were upregulated at 3D and 5D drought stress in the tolerant line but downregulated in the susceptible line (Table 4 and Table 7). In *Arabidopsis*, flowering-related genes such as GI and MFT were expressed earlier to accelerate flowering in response to drought stress [82]. *Arabidopsis* AATP1 genes were involved in seed maturation by influencing mitochondrial function under abiotic stress [83]. Collectively, FPF1 might have played a drought escape role via the promotion of flowering in the tolerant line and the prevention of premature interruption of inflorescence development. Moreover, the upregulation of AATP1 genes might have mediated the developmental and environmental signals in the tolerant line to maintain proper seed maturation process, contributing to plant fitness to energy and nutrient distributions during drought stress.

Transcription factors (TFs) of the family MYB, MYC, WRKY, bZIP, DREB (AP2/ERF), and NAC are widely known to respond to drought stress in plants [19,84]. In the current study, DREB1A (*Zm00001d036003*), MYB4 (*Zm00001d041853*), MYB63 (*Zm00001d002476*), ERF11 (*Zm00001d024324*) were all upregulated in the tolerant line at 3D drought stress and in both inbred lines at 5D drought stress (Table S4). Over-expression of DREB1A improves drought, freezing, and cold stress tolerance in transgenic plants through the accumulation of raffinose, galactinol, proline, and stress-inducible target genes such as early responsive to dehydration (ERD), cold-regulated (COR), and KIN genes [85]. In addition, GRF2 (*Zm00001d033876*), GLK44 (*Zm00001d034160*), and GLK41 (*Zm00001d026106*) TFs were downregulated at 5D drought stress in the tolerant line but upregulated in the susceptible line (Table 7). G2-like TFs plays a key role in chloroplast development and the expression of nuclear photosynthetic genes [86]. Photosynthesis was drastically affected in the susceptible line, and enhanced expression of GLK TFs might be related to its role in coordinating the expression of the photosynthetic apparatus. A bHLH103 (*Zm00001d033957*), VQ23 (*Zm00001d052220*), and CYCLOPS TFs (*Zm00001d045537*) were upregulated in the tolerant line at 3D and 5D drought stress, respectively (Table 4 and Table 7). The previous report of Wei et al. [87] highlighted that *ZmbHLH103* binds to the G-box element of downstream drought-responsive genes and regulate their transcriptions while CYCLOPS TF plays a significant role as a calcium sensor to bind to Ca^2+^ ions, thereby changing their conformations and functions, which might lead to drought tolerance [88]. Collectively, the differential expression of various TFs families such as the one named above and their interaction with each other in a complex network crucially contributes to drought stress tolerance.

Histones are the key chromatin proteins and the dynamic association of histones and their variants can regulate gene expression [89]. In this study, histone encoding genes (*Zm00001d042730* and *Zm00001d020584*) were enhanced in the susceptible line but suppressed in the tolerant line at 3D and 5D drought stress (Table 6 and Table 7). Abiotic stresses lead to DNA damage which must be repaired [90], and histone chaperones possess the capacity to modulate gene expression and DNA repair. Therefore, drought stress might have caused drastic DNA damage in the susceptible line, which led to the upregulation of repair machinery. The previous study in rice showed that a functional H3/H4 histone mediated abiotic stress adaptation by transcriptional regulation of diverse stress-related genes [91].

Plant abscisic acid stress ripening (ASR) induced proteins to impart tolerance to multiple abiotic stresses by regulating ABA biosynthesis, promoting stomatal closure, as well as acting as chaperone proteins [92,93]. AASR3 (*Zm00001d003712*), AASR5 (*Zm00001d025401*) encoding genes were upregulated at 3D and 5D, respectively, in the tolerant line but downregulated in the susceptible line (Table 4 and Table 5). Over-expression of *ZmASR3* improved drought stress tolerance in transgenic *Arabidopsis* plants by increasing stomata closure, improving the antioxidant system, regulating the ABA-dependent pathway, lowering MDA levels, and enhancing RWC and proline content [94]. A better understanding of the coordinated roles of these maize ASR genes in drought stress acclimation will be of paramount importance to maize breeders and researchers. Gibberellins (GAs) regulate various aspects of plant growth and development. GA2ox catalyzes bioactive GAs to inactive forms, thereby playing a key role in determining the levels of bioactive GAs. Therefore, upregulation of GA2ox8 (*Zm00001d002999*) and GA2ox7 (*Zm00001d038695*) genes in the susceptible line (Table 6 and Table 7) suggests a reduced number of bioactive GAs levels which repress growth for drought stress adaptation.

Recently, a growing body of evidence suggests that lncRNAs play significant roles in the regulation of various biological processes, including plant growth and development, epigenetic responses, and the responses to various stresses [95,96]. In this study, 2613 high-confidence drought-responsive lncRNAs were identified, among which 284 were previously characterized as drought-responsive lncRNAs in maize by Zhang et al. [37] (Table S2). Moreover, 144 drought-responsive lncRNAs were homologous to previously identified lncRNAs by Boerner et al. [38] (Table S3). A report by Chung et al. [97] identified 98 drought-responsive lncRNAs in rice while 521 lncRNAs were identified in tomato leaves exposed to drought stress [98]. The maize genome is replete with transposable elements (TEs), and a large proportion of lncRNAs are either derived TEs or contain remnants of TEs [99]. In this study, we found that 573 drought-responsive lncRNAs were derived from TEs (TE-lncRNAs). Our results suggest that TE-lncRNAs might have played key regulatory roles in moderating drought stress responses of the two inbred lines. Yuanda et al. [99] identified 1077 differentially expressed lncRNAs transcripts, including 509 TE-lncRNAs in maize under abiotic stresses such as heat, cold, salt, and drought. Additional work is required to uncover the regulatory mechanisms and the functions of these drought-responsive lncRNAs, including TE-lncRNAs, in response to drought stress. Nonetheless, our intriguing result is consistent with previous findings showing that most lncRNAs are derived from TEs [100] and that lncRNAs and TE-lncRNAs play a significant role in drought stress response [99].

**Figure 8:**
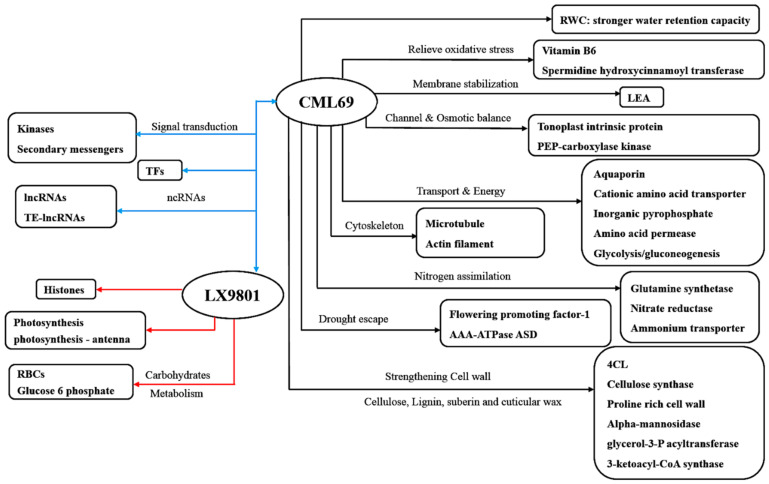
The schematic molecular model describing the main pathways involved in the acquisition of drought tolerance in maize seedling. The model was constructed based on our main common drought-responsive genes identified in this report, as well as plant abiotic stress pathway schemes previously described. The black and red pointing arrows display the main pathways which were enriched in CML69 and LX9801, respectively. The blue pointing arrows display the common pathways in both drought-tolerant and sensitive inbred lines. Abbreviation’s key: RWC, relative water content; LEA, late embryogenesis abundant; CL, coumarate-CoA ligase; TFs, transcription factors; ncRNAs, noncoding RNAs; lncRNAs, long noncoding RNAs; TE-lncRNAs, transposable elements long noncoding RNAs; RBCs, ribulose carboxylase.

Based on our key findings of the common drought-responsive DEGs and their associated pathways, in addition to the relevant published citations contained in this study, we developed a molecular model for drought stress tolerance in maize seedlings, as shown in Figure 8.

## 4. Materials and Methods

### 4.1. Plant Materials, Growth Conditions, and Drought Stress Treatments

Two maize (*Zea mays* L.) inbred lines with contrasting drought responses (tolerant CML69 and sensitive LX9801) were used in this experiment. The two inbred lines were obtained from a natural variation panel of maize, and their resistance to drought stress has been evaluated by a previous study [101,102]. After being soaked with water at 28 °C for 36 h, plump seeds were selected and sown in 44 × 28 × 8 cm rectangular boxes (twenty-five plants for each inbred line) filled with a mixture of vermiculite, nutrient soil, and garden soil (1:1:1) at 28 ± 2 °C room temperature with a 16-h light/8-h dark photoperiod. When the third leaves were fully expanded, the leaves tissues of the two inbred lines were harvested and samples labeled as control (C). The two inbred lines’ seedlings were then subjected to natural drought stress conditions by withholding water for three and five days. The samples collected three days post-drought treatment exposure were labeled 3D, while those collected five days post-drought treatment were labeled 5D for transcriptomic analysis and physiological assays. All samples were immediately frozen in liquid nitrogen and stored at −80 °C prior to subsequent analyses. All the samples were in three biological replicates making a total of 18 samples. In addition, four plants (two plants from each inbred line) were grown in 10 × 10 × 8 cm pots to show their phenotypic responses to drought stress. In this study, we decided to use one C per inbred line because the drought resistance of the two inbred lines we used had already been established, and the main goal of our work was to identify the differentially expressed genes between the two lines and reveal the potential molecular mechanisms. Several researchers have already exploited this element of using a single control in drought and salinity stress studies in contrast to distinct time-point treatments [103,104,105].

### 4.2. Physiological and Phenotypic Characterizations

The relative electrolyte leakage (REL) was determined as described by Blum et al. [106]. The leaves were washed three times with sterilized deionized water. Twenty leaf segments (0.8 cm in diameter) were vacuumized for 10 min in tubes containing 10 mL of sterilized deionized water and incubated at 25 °C for 1 h, and then the electrical conductivity (L_1_) was measured. Subsequently, the tubes were autoclaved at 100 °C for 10 min and then cooled to 25 °C before the final electrical conductivity (L_2_) was measured. The REL was calculated by the formula:

REL (%) = (L_1_ − L_0_)/(L_2_ − L_0_) × 100 (L_0_ = conductivity of deionized water).

Relative water content (RWC) was measured as described by Galmés et al. [107]. A total of 1 g of fresh leaves was cut into 1 cm fragments and weighed for the determination of fresh weight (W_f_). Then, put the leaves in pure water for 6 h, and measure the weight when the leaves are saturated (W_t_). The leaves were finally oven-dried to a constant dry weight (W_d_) for 10 h at 80 °C. RWC was measured according to the formula:

RWC (%) = (W_f_ − W_d_)/(W_t_ − W_d_) × 100

The thiobarbituric acid (TBA) method [108] was used to determine the malondialdehyde (MDA) content with some modifications. A total of 0.5 g of frozen sample was placed in a mortar, and 2 mL of pre-cooled phosphate buffer (pH = 7.8) was added. The leaves were ground into a homogenate, diluted to 5 mL (V), and transferred to a centrifuge tube at 4500 rpm for 10 min at 4 °C. A total of 1 mL of the supernatant (V2) and 2 mL 5% trichloroacetic acid solution containing 0.5% (w/v) TBA (V1) were heated at 100 °C for 10 min and then rapidly cooled in an ice bath. After centrifugation at 45,000 rpm for 10 min, the supernatant absorbance was measured at 532 nm (A_532_) and 600 nm (A_600_), respectively. MDA was calculated by the formula:

MDA [nmol/g (FW)] = [(A_532_ − A_600_) × V1 × V]/1.55 × 10^−1^ × FW × V2

### 4.3. Total RNA Extraction, cDNA Library Construction and Transcriptome Sequencing

Total RNA of the leaf samples (control and 3D, 5D- drought exposed leaf samples of both inbred lines) was isolated using the TRIzol reagent (Invitrogen, San Diego, CA, USA) according to the manufacturer’s protocol. The RNA quality was monitored on 1% agarose gels while the RNA concentration was checked using the NanoDrop 1000 spectrophotometer (NanoDrop Technologies Inc., Wilmington, DE, USA). The RNA integrity was further checked using the Agilent 2100 Bioanalyzer (Agilent Technologies, Santa Clara, CA, USA). Epicentre Ribo-zero™ rRNA Removal Kit (Illumina, San Diego, CA, USA) was used to remove ribosomal RNA (rRNA), and the cDNA library preparations of the 18 samples were constructed using a NEBNext^®^ Ultra™ Directional RNA Library Prep Kit for Illumina^®^ (NEB, Lpswich, MA, USA) following the manufacturer’s protocol. All the cDNA libraries were then sequenced on the Illumina HiSeq™ 2500 platform (Illumina, San Diego, CA, USA).

### 4.4. Sequencing Reads Processing, Mapping, and Gene Expression Quantification

The raw sequence reads we obtained via Illumina HiSeq™ 2500 platform were pre-processed with the Fastx-toolkit pipeline (http://hannonlab.cshl.edu/fastx_toolkit/index.html) (accessed on 15 January 2018). This was followed by trimming the raw data by removing the reads containing adaptors and low-quality sequences. The Phred quality scores, including Q20 (99% base call accuracy), Q30 (99.9% base call accuracy), as well as the GC content and sequence duplication level of the clean data, were calculated. Consequently, high-quality clean data was used in all the subsequent analyses. The B73_v4 reference genome was downloaded from the maize genome database (https://www.maizegdb.org/genome/genome_assembly/Zm-B73-reference-gramene-4.0) (accessed on 8 March 2018). All the clean reads obtained from the 18 samples were aligned to the reference genome using HISAT2 software [109] with default parameters (accessed on 6 September 2018). Then the aligned reads were assembled into transcripts, and the transcripts from all samples were merged using Cufflinks [36] (accessed on 6 September 2018). The assembled transcripts were compared to the reference annotation by Cuffcompare (accessed on 6 September 2018). The differential expression analysis of genes and transcripts was performed using Cuffdiff, which calculated the FPKM (fragments per kilobase of exon per million fragments mapped) of each gene on the 18 samples of CML69 and LX9801 (accessed on 6 September 2018). A transcript was considered differentially expressed if the log2 foldchange between control and stressed samples was equal or greater than 1 or less than −1, a *p*-value is less than 0.05, and nucleotide length is greater or equal to 200 bp. Differential expression analysis was graphically presented by the CummeRbund package (accessed on 15 September 2018) (http://www.bioconductor.org/packages/release/bioc/html/cummeRbund.ht-ml).

### 4.5. Functional Annotation of Gene Transcripts

For functional annotation, the 10,084 transcripts, which qualified to be our differentially expressed genes (DEGs), were annotated against maize genome B73_v4 (http://ensembl.gramene.org/Zea_mays/Info/Index) (accessed on 15 October 2018). In total, 6906 (68.5%) DEGs were annotated, and 3178 DEGs were unannotated. To elucidate the function of the 3178 unannotated genes, we applied the following procedures to identify high-confidence long noncoding RNAs (lncRNAs); (i) unannotated DEGs lengths were confirmed to be longer than 200 nucleotides for further analysis; (ii) DEGs that encode open reading frames (ORFs) of 120 or fewer amino acids were retained as lncRNA candidates; (iii) DEGs with similarity to known proteins based on BlastX against Swiss-Prot database were filtered out; (iv) all the 3178 unannotated DEGs were further evaluated using Coding Potential Calculator 2 (CPC 2.0) (http://cpc2.cbi.pku.edu.cn) [110] (accessed on 10 November 2018), which assesses the coding probability of transcripts; (v) a total of 2613 high-confidence drought-responsive lncRNAs were obtained by comparing the output of the two procedures. The sequences of the 2613 potential lncRNAs were aligned against previously reported drought-responsive lncRNAs and lncRNAs in maize using BlastX (E-value ≤0.001, homology ≥90%, and coverage ≥80%). All the 2613 potential lncRNAs were run through RepeatMasker (www.repeatmasker.org) for repetitive element content analysis (accessed on 20 December 2018).

### 4.6. Gene Ontology (GO) Enrichment and KEGG Pathway Enrichment Analyses

The biological enrichment of the DEGs was conducted by agriGOv2 (http://systemsbiology.cau.edu.cn/agriGOv2/) [111] (accessed on 20 October 2018). Significant enriched GO terms were determined by the *p*-value ≤ 0.05 with the Fisher’s exact test and the Bonferroni multi-test adjustment. Redundant GO terms were removed using Revigo [112]. Significantly enriched GO terms were assigned to the GO categories of biological process (BP), molecular function (MF), and cellular component (CC). The KEGG (http://www.genome.jp/kegg/) (accessed on 20 October 2018) [113] database was used to analyze the functional involvement of DEGs in various metabolic pathways. Moreover, KOBAS 3.0 webserver (http://kobas.cbi.pku.edu.cn/wait_kobas.php) [114] was used to test the statistical enrichment of DEGs in KEGG pathways (accessed on 20 October 2018). A *p*-value ≤ 0.05 was the threshold for significantly enriched KEGG pathways.

### 4.7. Quantitative Real-Time (qRT-PCR) Analysis

To validate the repeatability of the RNA-seq data, ten DEGs were randomly selected for verification by qRT-PCR. The operation procedure was similar to as previously described by Yu et al. [51]. Briefly, the RNA samples subjected to RNA-seq were also used for qRT-PCR, and the total RNA was purified with RNase-free DNase (Invitrogen, Gaithersburg, MD, USA) following the synthesizing of single-stranded cDNA using recombinant M-MLV reverse transcriptase (Invitrogen) according to the manufacturer’s protocol. The gene-specific primers (Table S12) used for qRT-PCR analysis were designed using Primer Premier 5.0 software (Premier Biosoft International, Palo Alto, CA, USA). The internal reference glyceraldehyde-3-phosphate dehydrogenase (GAPDH) was used to normalize the expression data. Relative expression levels were calculated according to the 2^−ΔΔCT^ (cycle threshold) method [115].

### 4.8. Statistical Analysis of Physiological Data

The SPSS software (version 19.0; SPSS Institute Ltd., Armonk, NY, USA) was used to conduct the analysis of variance for the physiological data. Fisher’s protected least significant differences (PLSD) test was used to separate means significant effect at *p* ≤ 0.05.

## 5. Conclusions

In this study, we comprehensively compared the leaf transcriptome and physiological responses of drought-tolerant (CML69) and drought-susceptible (LX9801) maize inbred lines at the seedling stage after three and five days of drought exposure. Resultantly, the tolerant-line seedlings maintained comparatively higher relative water content but lower relative electrolyte leakage and MDA content than the susceptible line. Using an RNA-seq-based approach, we mined out two critical sets of drought-responsive DEGs, including 4687 genotype-specific responsive genes and 2219 common drought-responsive genes. The latter indicates the existence of conserved drought-induced regulation pathways between the two maize genotypes. Among the 2219 DEGs, 84 and 315 DEGs were exclusively upregulated at 3D drought stress in the tolerant and susceptible lines, respectively. In addition, 73 and 43 DEGs were upregulated at 5D drought in the tolerant and susceptible lines, respectively. The DEGs of the drought-stressed tolerant line were predominantly associated with the cytoskeleton, cell wall modification, glycolysis/gluconeogenesis, transport, osmotic regulation, drought avoidance, ROS scavengers, defense, and transcriptional factors such as DREB1A, CYCLOP, bHLH, and G2-like. Contrary, the susceptible-line DEGs were enriched in photosynthesis, histone, and carbohydrate metabolism. The photosynthesis ability of the susceptible line was diminished as early as 3D of drought, as echoed by the downregulation of photosynthesis-related genes. The upregulation of histones encoding genes reflects the damage caused by drought in the susceptible line, while downregulation of carbon fixation genes such as Rubisco at 3D drought stress in the susceptible line implies that the efficiency of CO_2_ assimilation was suppressed, and carbon fixation was blocked. Nonetheless, lncRNAs, including TE-lncRNAs, played a significant role in the drought stress response of the two inbred lines. Our findings enhance further elucidation of the molecular networks mediating maize drought tolerance at the seedling stage as well as providing the invaluable foundational basis for future research based on downstream analysis of the identified specific individual genes.

## Figures and Tables

**Table 1:** Expression patterns of the 10,084 DEGs by inbred line and drought stress stage.

Inbred Line ^1^	Comparison ^2^	DEG Number ^3^	Upregulated ^4^	Downregulated ^5^
CML69	C vs. 3D	1902	745	1157
	C vs. 5D	5385	2511	2874
LX9801	C vs. 3D	3362	1321	2041
	C vs. 5D	5512	2434	3078

^1^ Inbred line, maize cultivars inbred lines; CML69 (drought-tolerant), LX9801 (drought-susceptible); ^2^ comparison, experimental comparison group; C = control, 3D = three days drought treatment, 5D = five days drought treatment; ^3^ DEG number, total number of differentially expressed genes (DEGs) in the group; ^4^ Upregulated, number of DEGs whose expression levels were increased; ^5^ Downregulated, number of DEGs whose expression levels were decreased by drought stress treatment.

**Table 2:** Expression patterns of the 6906 annotated DEGs by inbred line and drought stress stage.

Inbred Line ^1^	Comparison ^2^	DEG Number ^3^	Upregulated ^4^	Downregulated ^5^
CML69	C vs. 3D	1294	596	698
	C vs. 5D	4145	2154	1991
LX9801	C vs. 3D	2488	1094	1394
	C vs. 5D	3996	2160	1836

^1^ Inbred line, maize cultivars inbred lines; CML69 (drought-tolerant), LX9801 (drought-susceptible); ^2^ comparison, experimental comparison group; C = control, 3D = three days drought treatment, 5D = five days drought treatment; ^3^ DEG number, total number of differentially expressed genes (DEGs) in the group; ^4^ Upregulated, number of DEGs whose expression levels were increased; ^5^ Downregulated, number of DEGs whose expression levels were decreased by drought stress treatment.

**Table 3:** Expression of the 2219 common drought-responsive DEGs by inbred line and drought stress stage.

Inbred Line ^1^	Comparison ^2^	DEG Number ^3^	Upregulated ^4^	Downregulated ^5^
CML69	C vs. 3D	713	381	332
	C vs. 5D	2114	1105	1009
LX9801	C vs. 3D	1350	607	743
	C vs. 5D	2014	1072	942

^1^ Inbred line, maize cultivars inbred lines; CML69 (drought-tolerant), LX9801 (drought-susceptible); ^2^ comparison, experimental comparison group; C = control, 3D = three days drought treatment, 5D = five days drought treatment; ^3^ DEG number, total number of differentially expressed genes (DEGs) in the group; ^4^ Upregulated, number of DEGs whose expression levels were increased; ^5^ Downregulated, number of DEGs whose expression levels were decreased by drought stress treatment. The 3178 unannotated drought-responsive DEGs (≥200 bp) were uploaded to the CPC 2.0 website for classification as protein-coding or noncoding RNAs. A total of 587 DEGs were identified as potential coding RNAs, while 2591 were classified as long noncoding RNAs (lncRNAs) (Figure S2). Moreover, the 3178 unannotated DEGs were scanned for the open reading frame (ORF). A total of 1510 DEGs with an ORF greater than 120 amino acids (AA) were discarded. The remaining 1668 DEGs (ORF length ≤ 120 AA) were aligned to the Swissprot database to identify homologous proteins. A total of 177 DEGs were discarded after being homologous to known proteins (E-value ≤ 0.001), while the remaining 1491 DEGs were classified as lncRNAs (Figure S2). In totality, 2613 drought-responsive lncRNAs (Figure S2) were identified from 3178 unannotated DEGs implying the role of lncRNAs in the drought stress response of the two inbred lines.

**Table 4:** Sixteen drought-responsive DEGs upregulated in CML69 but downregulated in LX9801 at 3D drought stress.

Gene ID ^1^	Log2 Ratio (CML69_C vs. 3D) ^2^	Log2 Ratio (LX9801_C vs. 3D) ^3^	Best-Hit-A.th ^4^	Symbol ^5^	Annotation ^6^
Zm00001d003712	1.067078963	−1.903915782	-	AASR3	Abscisic acid stress ripening 3
Zm00001d004203	1.157175723	−2.539146896	AT5G24860.1	FPF1	Flowering promoting factor 1
Zm00001d042143	1.727547967	−2.192605603	AT3G57240.1	BG3	Beta−1,3-glucanase 3
Zm00001d008746	10.38729576	−1.248462466	AT5G09810.1	ACT7	Actin 7
Zm00001d013410	1.03408084	−1.525068282	AT3G12110.1	ACT11	Actin-11
Zm00001d011734	1.100182925	−2.061333851	-	-	Inorganic pyrophosphatase 1
Zm00001d024027	1.480081659	−1.011948016	-	PRCW1	Proline-rich cell wall protein
Zm00001d016301	1.822076998	−2.04090855	AT1G17710.1	PEPC1	Pyridoxal phosphate phosphatase
Zm00001d033385	1.290037527	−2.314823007	AT3G50760.1	GATL2	Galacturonosyltransferase
Zm00001d031875	1.621452385	−2.335532517	AT3G54700.2	PHT1;7	Phosphate transporter 1
Zm00001d032850	1.505609835	−1.263658023	AT2G38940.1	PHT1;2	Phosphate transporter 1
Zm00001d033241	1.03144486	−2.848924659	AT4G21120.1	CAT1	Cationic amino acid transporter
Zm00001d033957	1.211020519	−1.721699461	-	bhlh103	bHLH-transcription factor 103
Zm00001d038151	1.335042999	−1.682288928	AT1G03220.1	SAP2	Aspartic protease 2
Zm00001d044157	1.827511783	−1.875850961	AT3G14690.2	CYP72A15	Cytochrome P450
Zm00001d047436	1.44118319	−1.335352169	AT5G40010.1	AATP1	AAA-ATPase 1

^1^ Gene ID, unique gene identifying number in the Maize Genetic and Genomics Database (Maize GDB); ^2^ log2 ratio (CML69_C vs. 3D), fold change, calculated as the ratio of expression of upregulated or downregulated genes between drought stress and control of CML69, negative fold change means that the genes were downregulated while positive fold change value means that the genes were upregulated; ^3^ log2 ratio (LX9801_C vs. 3D), similar with number 2 but in LX9801 cultivar; ^4^ Best-hit-A.th, *Arabidopsis thaliana* gene, which is homologous to the maize Gene ID; ^5^ Symbol, scientific symbol of the *Arabidopsis thaliana* gene, hyphen (-) indicates that there was no symbol for that specific gene; ^6^ Annotation, description/function of the gene identified by the given Gene ID and Best-hit-A.th.

**Table 5:** Thirty-three drought-responsive DEGs upregulated in CML69 but downregulated in LX9801 at 5D drought stress.

Gene ID ^1^	Log2 Ratio (CML69_C vs. 5D) ^2^	Log2 Ratio (LX9801_C vs. 5D) ^3^	Best-Hit-A.th ^4^	Symbol ^5^	Annotation ^6^
Zm00001d007606	1.544362812	−2.045097787	AT5G41040.2	ASFT	Aliphatic Suberin feruloyl transferase
Zm00001d032103	1.862733313	−1.816378054	AT3G21240.1	4CL2	4-coumarate-CoA ligase
Zm00001d047972	1.17316282	−1.327960157	AT2G01900.1	t5ptase	Inositol polyphosphate 5-phosphatase
Zm00001d030285	1.064851308	−1.575733162	AT5G66150.2	-	Alpha-mannosidase
Zm00001d017494	1.745450662	−1.355124162	AT2G45110.1	EXPB4	Expansin B4
Zm00001d003091	2.535603442	−2.019264795	AT1G08280.1	GALT29A	Glycosyltransferase
Zm00001d011838	1.124191501	−1.027094089	AT2G41640.1	GALT61	Glycosyltransferase family 61
Zm00001d024687	1.155209579	−1.009500907	AT4G15240.1	GALT	Glycosyltransferase
Zm00001d052209	3.110619463	−1.221666072	AT1G22360.1	UGT85A2	UDP-glucosyl transferase
Zm00001d017249	1.211898981	−2.268909254	AT1G64780.1	AMT1;2	Aammonium transporter 1
Zm00001d018751	2.227663156	−2.263570515	AT5G49630.1	AAP6	Amino acid permease 6
Zm00001d035717	1.506215854	−1.568896164	-	WAT1	Walls are thin 1
Zm00001d037515	1.689789607	−1.669586632	AT5G01790.1	-	Hypothetical protein
Zm00001d025724	1.855649477	−1.13455352	AT1G67600.1	-	Acid phosphatase haloperoxidase
Zm00001d028367	1.2039208	−2.231720672	AT1G14700.1	PAP3	Purple acid phosphatase 3
Zm00001d017270	1.586319405	−1.991000978	AT3G04530.1	PPCK2	Phosphoenolpyruvate carboxylase kinase
Zm00001d037783	1.167986012	−1.248374599	AT3G23000.1	CIPK7	Serine/threonine protein kinase
Zm00001d050164	1.109395435	−2.580502154	-	WAKL20	Wall-associated receptor kinase-like 20
Zm00001d007773	1.373813395	−1.058825309	AT3G28510.1	AATP1	AAA-ATPase ASD mitochondrial
Zm00001d047436	1.511985756	−1.267657	AT5G40010.1	AATP1	AAA-ATPase 1
Zm00001d011734	2.245158893	−1.083662395	-	-	Inorganic pyrophosphatase 1
Zm00001d013565	4.481035303	−1.578440935	AT4G37810.1	EPFL2	Epidermal patterning factor
Zm00001d016269	1.238419504	−1.361567296	AT5G25840.1	DUF1677	DUF1677 family protein
Zm00001d016301	3.330549142	−2.375063337	AT1G17710.1	PEPC1	Pyridoxal phosphate phosphatase
Zm00001d016548	2.431505237	−1.14796758	AT1G67430.1	-	-
Zm00001d025401	1.538596646	−1.066777498	-	AASR5	Abscisic acid stress ripening 5
Zm00001d037797	1.469551335	−1.783796353	AT2G24000.2	SCPL22	Serine carboxypeptidase
Zm00001d048947	3.3420077	−1.714779709	AT3G04720.1	PR4	Pathogenesis-related 4
Zm00001d044541	1.502454541	−2.085986029	AT2G45130.1	SPX3	SPX domain protein 3
Zm00001d052220	2.229459236	−1.898568624	-	VQ23	VQ motif-transcription factor
Zm00001d045537	1.133943728	−2.756015494	-	-	CYCLOPS
Zm00001d043665	1.599461141	−1.494855603	-	-	-

^1^ Gene ID, unique gene identifying number in the Maize Genetic and Genomics Database (Maize GDB); ^2^ log2 ratio (CML69_C vs. 3D), fold change, calculated as the ratio of expression of upregulated or downregulated genes between drought stress and control of CML69, negative fold change means that the genes were downregulated while positive fold change value means that the genes were upregulated; ^3^ log2 ratio (LX9801_C vs. 3D), similar with number 2 but in LX9801 cultivar; ^4^ Best-hit-A.th, *Arabidopsis thaliana* gene, which is homologous to the maize Gene ID; ^5^ Symbol, scientific symbol of the *Arabidopsis thaliana* gene, hyphen (-) indicates that there was no symbol for that specific gene; ^6^ Annotation, description/function of the gene identified by the given Gene ID and Best-hit-A.th.

**Table 6:** Eleven drought-responsive DEGs downregulated in CML69 but upregulated in LX9801 at 3D drought stress.

Gene ID ^1^	Log2 Ratio (CML69_C vs. 3D) ^2^	Log2 Ratio (LX9801_C vs. 3D) ^3^	Best-Hit-A.th ^4^	Symbol ^5^	Annotation ^6^
Zm00001d002999	−1.586466311	1.125018924	AT4G21200.1	GA2OX8	Gibberellin 2-oxidase 8
Zm00001d012131	−1.32220827	1.195476431	AT3G62920.1	-	Zinc metalloproteinase
Zm00001d017666	−2.335920246	2.562937104	AT1G32450.1	NRT1.5	Nitrate transporter 1.5
Zm00001d021781	−3.589512662	1.006168604	AT1G79360.1	OCT2	Organic cation transporter 2
Zm00001d039886	−1.343911524	1.867730502	AT3G54110.1	PUMP1	Uncoupling protein PUMP2
Zm00001d042730	−1.845122758	2.690906476	AT5G65360.1	H3.1	Histone
Zm00001d020584	−10.83217007	10.97500245	AT5G59690.1	-	Histone
Zm00001d026106	−1.39634541	1.54543134	-	Glk41	G2-like-transcription factor 41
Zm00001d031673	−1.48213493	1.180787578	AT1G43160.1	RAP2.6	Related to AP2 6
Zm00001d039859	−1.141107245	1.392329759	AT3G09390.1	MT2A	Metallothionein
Zm00001d043795	−1.222220983	1.882006981	AT1G10360.1	GSTU18	Glutathione S-transferase TAU 18

^1^ Gene ID, unique gene identifying number in the Maize Genetic and Genomics Database (Maize GDB); ^2^ log2 ratio (CML69_C vs. 3D), fold change, calculated as the ratio of expression of upregulated or downregulated genes between drought stress and control of CML69, negative fold change means that the genes were downregulated while positive fold change value means that the genes were upregulated; ^3^ log2 ratio (LX9801_C vs. 3D), similar with number 2 but in LX9801 cultivar; ^4^ Best-hit-A.th, *Arabidopsis thaliana* gene, which is homologous to the maize Gene ID; ^5^ Symbol, scientific symbol of the *Arabidopsis thaliana* gene, hyphen (-) indicates that there was no symbol for that specific gene; ^6^ Annotation, description/function of the gene identified by the given Gene ID and Best-hit-A.th.

**Table 7:** Thirty drought-responsive DEGs downregulated in CML69 but upregulated in LX9801 at 5D drought stress.

Gene ID ^1^	Log2 Ratio (CML69_C vs. 5D) ^2^	Log2 Ratio (LX9801_C vs. 5D) ^3^	Best-hit-A.th ^4^	Symbol ^5^	Annotation ^6^
Zm00001d048709	−1.568831793	2.066012833	-	BX1	Benzoxazinless 1
Zm00001d048710	−3.193078195	1.516375732	-	BX2	Benzoxazinone synthesis 2
Zm00001d048702	−2.089702354	1.157137323	-	BX3	Benzoxazinone synthesis 3
Zm00001d018206	−2.406569277	1.214850007	AT1G37130.1	NR2	Nitrate reductase 2
Zm00001d033747	−1.076317875	1.504587352	AT5G37600.1	GSR 1	Glutamine synthetase
Zm00001d043374	−1.516339733	1.240722383	AT1G52190.1	NRT1.1	Nitrate transporter
Zm00001d017666	−2.247773905	1.220192446	AT1G32450.1	NRT1.5	Nitrate transporter 1.5
Zm00001d026131	−1.101040846	1.057095664	-	LHT	Lysine histidine transporter-like 7
Zm00001d051362	−1.056763575	1.205752805	AT4G17340.1	TIP2	Tonoplast intrinsic protein 2
Zm00001d021653	−2.03788204	1.009690798	AT1G61800.1	GPT2	Glucose−6-phosphate translocator 2
Zm00001d018056	−1.257508316	1.100290806	AT1G26945.1	bHLH30	bHLH-transcription factor 30
Zm00001d026106	−2.455075759	1.96335238	-	Glk41	G2-like-transcription factor 41
Zm00001d033876	−1.529425044	1.281885695	AT4G37740.1	GRF2	Growth-regulating factor 2
Zm00001d034160	−1.128547313	1.256333329	-	glk44	G2-like-transcription factor 44
Zm00001d006857	−11.32647816	3.296768997	AT1G20950.1	-	Phosphofructokinase family
Zm00001d016444	−1.518798472	1.235047417	-	-	-
Zm00001d020584	−10.83217007	11.67180641	AT5G59690.1	-	Histone
Zm00001d020726	−1.804385564	2.404793087	AT1G47380.1	PP2C	Protein phosphatase 2C
Zm00001d021168	−1.22761115	2.022306741	AT2G43840.1	UGT74F1	UDP-glycosyltransferase 74 F1
Zm00001d025947	−1.138375432	1.60173917	AT3G60690.1	SAUR59	SAUR-like auxin-responsive
Zm00001d027861	−1.422607421	1.729671881	AT4G39660.1	AGT2	Alanine: glyoxylate aminotransferase
Zm00001d028693	−1.639385828	1.098782465	-	Saf1	Safener induced 1
Zm00001d030348	−1.953534228	1.091478477	AT1G64710.1	-	Alcohol dehydrogenase
Zm00001d034788	−1.200633487	1.688351727	AT1G77280.2	-	Kinase protein
Zm00001d036989	−1.501992276	1.044909155	AT4G37820.2	-	Transmembrane protein
Zm00001d038209	−1.16700922	1.0113365	AT5G49820.1	RUS6	Root UVB sensitive protein
Zm00001d038695	−1.789903087	2.114447302	-	GA2ox7	Gibberellin 2-oxidase
Zm00001d039859	−1.549403992	2.883195042	AT3G09390.1	MT2A	Metallothionein
Zm00001d050694	−1.052286015	2.30335502	AT1G43710.1	EMB1075	Pyridoxal phosphate (PLP)
Zm00001d052651	−1.629711466	2.143596379	AT5G13870.3	XTH5	Xyloglucan endotransglucosylase

^1^ Gene ID, unique gene identifying number in the Maize Genetic and Genomics Database (Maize GDB); ^2^ log2 ratio (CML69_C vs. 3D), fold change, calculated as the ratio of expression of upregulated or downregulated genes between drought stress and control of CML69, negative fold change means that the genes were downregulated while positive fold change value means that the genes were upregulated; ^3^ log2 ratio (LX9801_C vs. 3D), similar with number 2 but in LX9801 cultivar; ^4^ Best-hit-A.th, *Arabidopsis thaliana* gene, which is homologous to the maize Gene ID; ^5^ Symbol, scientific symbol of the *Arabidopsis thaliana* gene, hyphen (-) indicates that there was no symbol for that specific gene; ^6^ Annotation, description/function of the gene identified by the given Gene ID and Best-hit-A.th.

## Data Availability

This manuscript includes the essential data either as figures or as supplementary materials. The raw sequence reads have been deposited to the Genome Sequence Archive (GSA) under accession numbers CRA003679 (https://ngdc.cncb.ac.cn/bioproject/browse/PRJCA004124) (accessed on 8 January 2021).

## Supplementary Materials

The following are available online at https://www.mdpi.com/article/10.3390/ijms22136980/s1, Figure S1: Correlation of the expression values of the biological replicates of both samples, Figure S2: Analysis of 3178 unannotated drought-responsive DEGs, Figure S3: Analysis of 2613 drought-responsive long noncoding RNAs (lncRNAs), Table S1: Summary of RNA sequencing results for the eighteen maize seedling leaf samples, Table S2: 284 lncRNAs are homologous to known drought-responsive lncRNAs in maize, Table S3: 144 lncRNAs are homologous to known lncRNAs in maize, Table S4 Drought-responsive genes upregulated in CML69 with no changes observed in LX9801 after 3D of drought stress treatment, Table S5: Drought-responsive genes downregulated in CML69 with no changes observed in LX9801 after 3D of drought stress treatment, Table S6: Drought-responsive genes upregulated in CML69 with no changes observed in LX9801 after 5D of drought stress treatment, Table S7: Drought-responsive genes downregulated in CML69 with no changes observed in LX9801 after 5D of drought stress treatment, Table S8: Drought-responsive genes upregulated in LX9801 with no changes observed in CML69 after 3D of drought stress treatment, Table S9: Drought-responsive genes downregulated in LX9801 with no changes observed in CML69 after 3D of drought stress treatment, Table S10: Drought-responsive genes upregulated in LX9801 with no changes observed in CML69 after 5D of drought stress treatment, Table S11: Drought-responsive genes downregulated in LX9801 with no changes observed in CML69 after 5D of drought stress treatment, Table S12: The primers of ten differentially expressed genes used for qRT-PCR verification.

## Abbreviations

DEGs	Differentially expressed genes
KEGG	Kyoto Encyclopedia of Genes and Genomes
GO	Gene ontology
LEA	Late embryogenesis abundant proteins
ROS	Reactive oxygen species
AQPs	Aquaporins
GAs	Gibberellins
ASR	Abscisic acid stress ripening
PEPC	Phosphoenolpyruvate carboxylase 1
Pi	Inorganic phosphate
C	Control
3D	Three days of drought stress
5D	Five days of drought stress
TFs	Transcription factors
MDA	Malondialdehyde
REL	Relative electrolyte leakage
BP	Base pairs
Vs.	Versus
AA	Amino acid
Rep	Replicates
lncRNA	Long noncoding RNA
TE-lncRNA	Transposable elements long noncoding RNA
