# Supplementary material for: Transcriptome Analysis of Tolerant and Susceptible Maize Genotypes Reveals Novel Insights about the Molecular Mechanisms Underlying Drought Responses in Leaves
Source: Int J Mol Sci. 2021 Jun 29;22(13):6980. doi: 10.3390/ijms22136980 (PMC8268334; doi:10.3390/ijms22136980)
Supplement: Supplementary file 1 [file ijms-22-06980-s001.zip › Supplementary figures.pdf]

International Journal of *Molecular Sciences*

MDPI

Article

Transcriptome Analysis of Tolerant and Susceptible Maize Genotypes Reveals Novel Insights about the Molecular Mechanisms Underlying Drought Responses in Leaves

Joram Kiriga Waititu [1,†], Xingen Zhang [2,†], Tianci Chen [2], Yang Zhao [2,*] and Huan Wang [1,3,*]

1 Biotechnology Research Institute, Chinese Academy of Agricultural Sciences, Beijing 100081, China; joram.kiriga@gmail.com
2 National Engineering Laboratory of Crop Stress Resistance Breeding, School of Life Sciences, Anhui Agricultural University, Hefei 230036, China; mubzs9r@qq.com (X.Z.); 1415987723@qq.com (T.C.)
3 National Agricultural Science and Technology Center, Chengdu 610213, China
† These authors contributed equally to this work.
* Correspondence: zhaoyang0521@163.com (Y.Z.); wanghuan@caas.cn (H.W.)

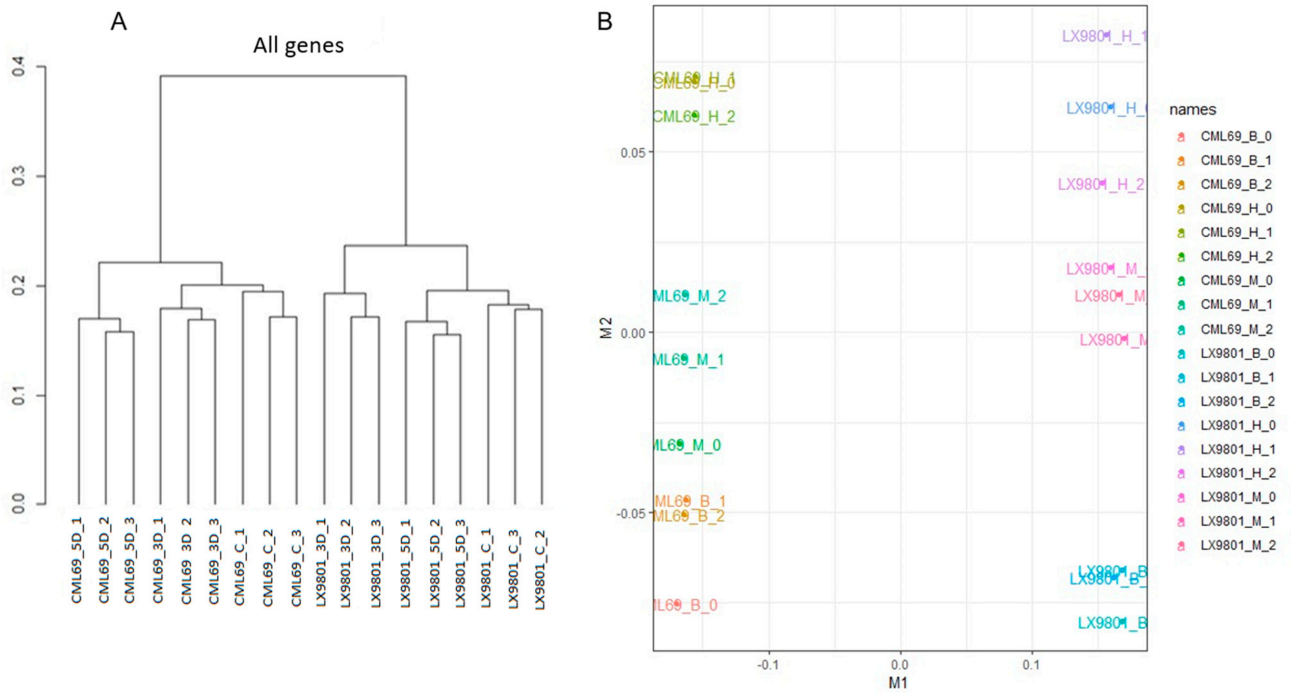

Figure S1. Correlation of the expression values of the biological replicates of both samples. (**A**) Dendrogram depicting correlation among replicates of the same sample on global expression profiles at C, 3D, and 5D drought stress. (**B**) Multidimensional scaling (MDS) plot analysis of the sample gene expression profiles. Each genotype was categorically grouped according to the similarity of individual replicates. B represents control (C), M represents three days (3D), while H represents five days (5D) drought treatment. 0, 1, 2 illustrates individual biological replications of the samples.

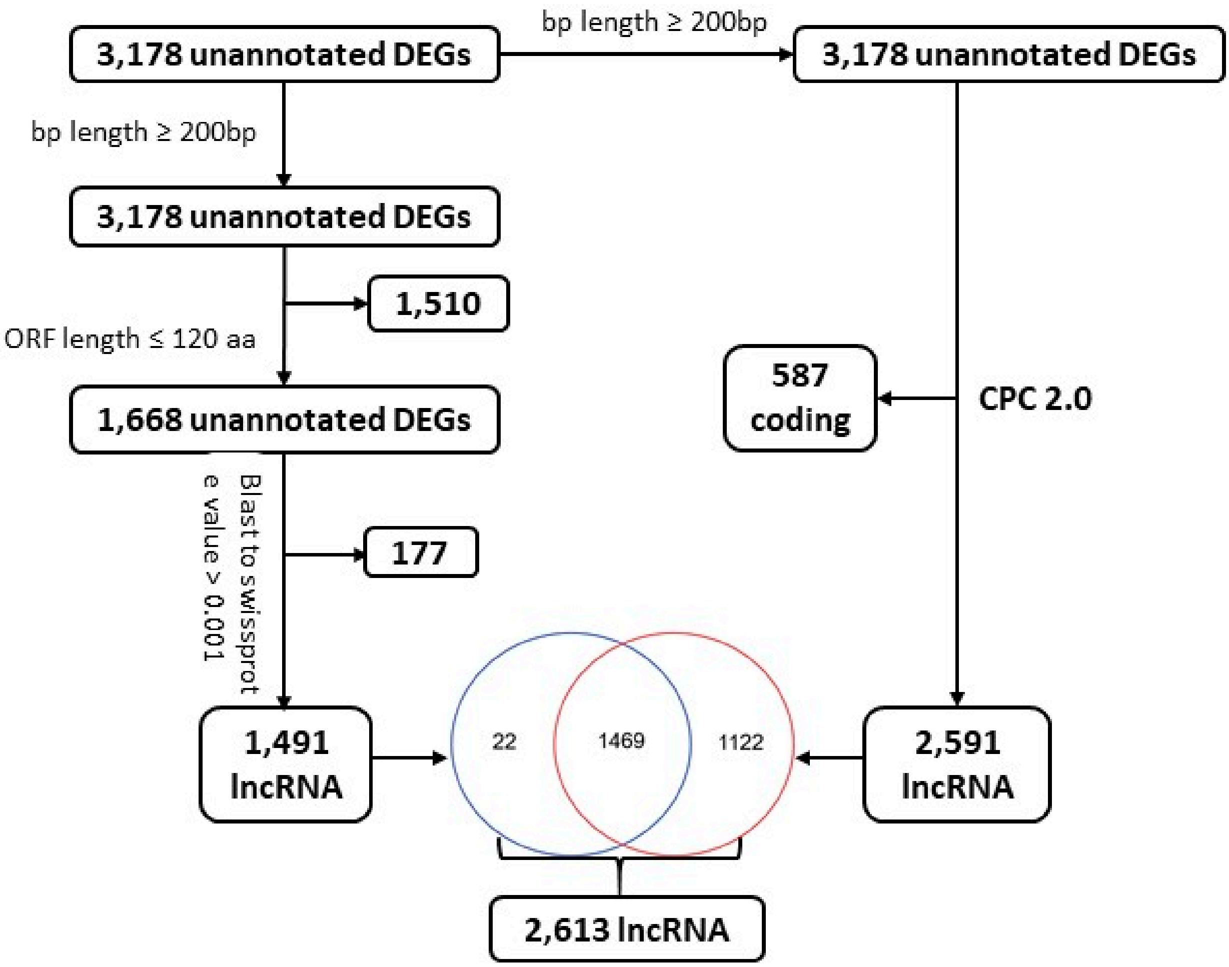

Figure S2. Analysis of 3,178 unannotated drought-responsive DEGs. CPC 2.0 generated 587 coding and 2,591 long non-coding RNAs. The ORF method generated 1,491 long non-coding RNAs. In totality, 2,613 out of 3,178 were long non-coding RNAs.

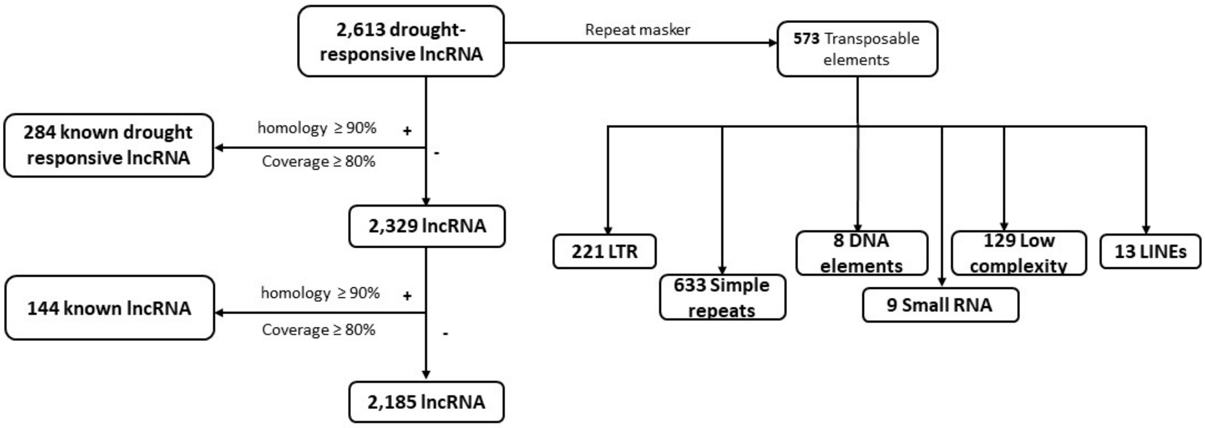

Figure S3. Analysis of 2,613 drought-responsive long non-coding RNAs (lncRNAs). 428 (284+144) are homologous to previously reported lncRNAs. 573 lncRNAs were identified to be Transposable elements (TEs) by RepeatMasker.
